# Recent advances and future directions on GLA-producing organisms

**DOI:** 10.3389/fbioe.2025.1567840

**Published:** 2025-07-09

**Authors:** Mojgan Latifi, Foroozan Jalali Bidgoli, Helia Hajihassani, Danial Hassani, Pär K. Ingvarsson, Naser Farrokhi

**Affiliations:** ^1^ Department of Cell & Molecular Biology, Faculty of Life Sciences and Biotechnology, Shahid Beheshti University, Tehran, Iran; ^2^ School of Food and Pharmacy, Institute of Biomaterials and Biomedicine, Shanghai Zhongqiao Vocational and Technical University, Shanghai, China; ^3^ Department of Plant Biology, Swedish University of Agricultural Sciences, Uppsala, Sweden

**Keywords:** gamma-linolenic acid, GLA production enhancem, metabolic engineering, genetic modification, genome editing technologies, GLA-producing microorganisms, omega-6 polyunsaturated fatty acids (PUFAs)

## Abstract

Gamma-linolenic acid (GLA) is a biologically active omega-6 fatty acid with anti-inflammatory, immunomodulatory, and cardiovascular protective effects. It is a vital constituent of human health and is finding more widespread applications in nutritional supplements, medications, and functional foods. GLA can be derived from many different natural sources, including plants, fungi, and microorganisms. This review paper presents an overview of the current advances in the discovery, metabolic engineering, and GLA-producing organism optimization. We further present a discussion on new biotechnological approaches—such as culture medium optimization, genetic engineering, and genome editing—that can be employed to enhance GLA production. The paper also presents new trends and directions in the commercial exploitation of GLA-containing products, unveiling new, health-oriented applications.

## Graphical Abstract

**Figure:**
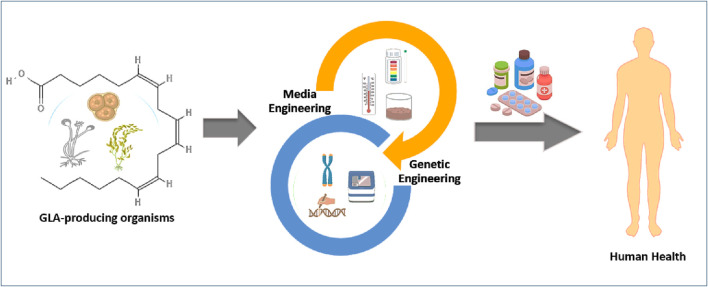
Organisms that produce GLA can be enhanced through media and genetic engineering techniques to develop better pharmaceuticals and supplements for humans.

## 1 Introduction

Gamma-linolenic acid (GLA) is a crucial omega-6 polyunsaturated fatty acid (PUFA) with significant implications for human health and nutrition. It is primarily found in seed oils from plants like *Ribes nigrum* (blackcurrant), *Borago officinalis* (borage), and *Cannabis sativa* (hemp). Structurally, GLA consists of an 18-carbon chain with three double bonds at the sixth, ninth, and 12th positions. As a precursor in the biosynthesis of other essential long-chain omega-6 fatty acids, such as dihomo-gamma-linolenic acid (DGLA) and arachidonic acid (ARA), GLA plays a pivotal role in maintaining various physiological processes. However, due to inefficiencies in the conversion of linoleic acid (LA) to GLA in some individuals, it is necessary to obtain GLA from dietary sources (Buist, 2010; Cunnane, 2003).

Beyond its fundamental nutritional value, GLA also possesses bioactive properties. The body can convert GLA into bioactive compounds such as DGLA, which exhibit both anti-inflammatory and potential anticancer effects *in vitro* (Van Hoorn et al., 2008). This conversion has led to studies exploring GLA supplementation as an alternative to nonsteroidal anti-inflammatory drugs (NSAIDs) for treating conditions like rheumatoid arthritis (Van Hoorn et al., 2008). Additionally, GLA has demonstrated potential benefits in areas such as cardiovascular health, diabetic neuropathy management, treatment of atopic dermatitis, and alleviation of cyclical mastalgia (Belch et al., 1988). Furthermore, GLA is being investigated as a potential therapeutic agent in the treatment of various cancers, including prostate, colorectal and pancreatic cancers (Kairemo et al., 1998; Kalampounias et al., 2024a; Kalampounias et al., 2024b). While most of the evidence remains confined to *in vitro* studies, these studies have shown promising results, although further clinical trials and epidemiological research are still required. Recent research also highlights that deficiencies in fatty acids, such as GLA, DGLA, ARA, EPA, and ALA may increase susceptibility to diseases like COVID-19. Milder cases of the virus have been observed in children and premenopausal women, who tend to have higher levels of beneficial fatty acids such as GLA and DGLA, which possibly help combat the virus (Das, 2021). This emphasises the unique role of GLA in the immune system, distinguishing it from other fatty acids and showcasing its potential impact on disease prevention.

Given its wide array of health benefits, the commercial market of GLA has experienced significant growth. In 2023, the global market for GLA was valued at US$ 56 million, with projections estimating it will reach US$ 71 million by 2030, reflecting a compound annual growth rate (CAGR) of 3.4% from 2024 to 2030 (Das, 2021). This growth highlights the importance of developing efficient and scalable production techniques, particularly those that incorporate advancements in genetic and metabolic engineering. Overcoming the challenges of scaling up metabolic engineering for GLA biomanufacturing will be essential for meeting future demand and ensuring a reliable, sustainable supply of this valuable compound.

As the scientific understanding of GLA’s health-promoting effects continues to grow, there is an escalating demand for its production, particularly within the pharmaceutical and nutraceutical sectors. Since the human body cannot synthesise GLA, it must be obtained through diet or supplementation (Mudronová et al., 2018). Traditional techniques, such as extracting GLA from plant seeds, often yield limited results and are not always cost-effective. To address these challenges, biomanufacturing approaches, particularly those employing genetic and metabolic engineering, have emerged as more sustainable and efficient alternatives. These biotechnological methods enable higher GLA yields while overcoming the limitations associated with traditional extraction procedures discussed above (Chutrakul et al., 2016).

## 2 Methodology

This comprehensive review examines the characteristics, biosynthetic pathways, and biotechnological advances of gamma-linolenic acid (GLA)-producing organisms with special reference to microbial and engineered systems. The review followed stringent PRISMA standards of transparency, methodological rigour, and reproducibility in the process of literature screening and analysis.

### 2.1 Database search and selection strategy

An extensive and systematic search was done across eight major scientific databases: PubMed, Scopus, Web of Science, ScienceDirect, Wiley, Taylor and Francis, SpringerLink, and Google Scholar. Boolean operators were used in combination with suitable keywords such as “gamma-linolenic acid”, “GLA-producing organisms”, “polyunsaturated fatty acid”, “health applications”, and “production methods” to isolate the most relevant articles.

### 2.2 Inclusion and exclusion criteria

The first search produced 4,493 documents. 1,114 articles were selected for full-text analysis after duplicates were removed and abstracts were screened for relevance. To determine relevance and scientific merit, the following inclusion criteria were applied: original research articles published between 2011 and 2024. English-language papers, and those concerned with GLA-producing organisms (microbial, algal, plant-derived, or engineered systems), direct investigation of biosynthesis pathways, production technology, or therapeutic applications. Exclusion criteria were as follows: non-research content such as news accounts, reviews, and editorials, articles with no full-text access, studies not directly applicable to GLA production or utilisation.

### 2.3 Data extraction and quality appraisal

Following the full-text screening, 190 articles were screened for thematic synthesis. Studies were thoroughly examined for study design, objectives, method, statistical quality, and direct applicability for GLA production or utilisation. Quality appraisal was conducted using the PRISMA checklist and the Cochrane risk-of-bias tool to select only high-quality evidence-based studies. The retrieved information was organised under the following focus categories: biosynthesis routes and metabolic engineering, genetic engineering platforms for higher GLA yields, extraction and purification technologies for GLA purification, commercial trends, market uses, and scalability.

### 2.4 Bibliometric analysis and thematic trends

**FIGURE 1:**
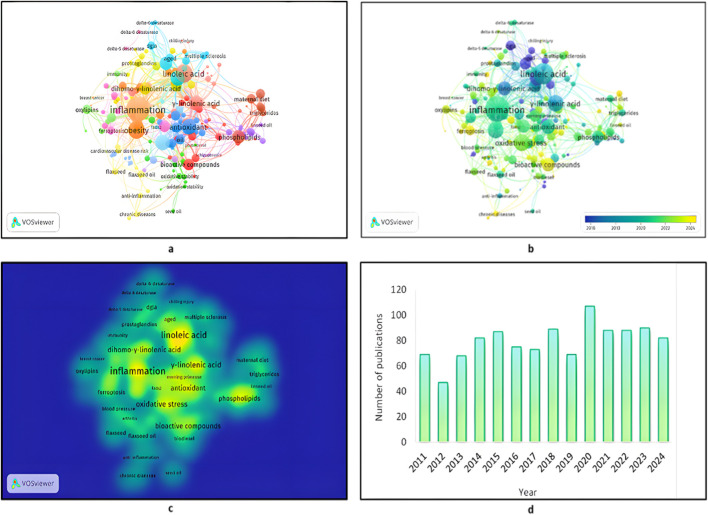
The most frequently occurring keywords in GLA-related research articles. **(a)** Keyword co-occurrence network of the relationship between GLA, DGLA, LA, and associated biological processes. **(b)** Clustered visualisation of similar keywords describing research themes such as inflammation, metabolic disorder, and oxidative stress. **(c)** Frequency of the most frequently used keywords among studies under study, determining research focus. **(d)** Research article count in relation to different topics in GLA. The keyword analysis was performed employing VOSviewer (version 1.6.20) with a binary counting method and a minimum threshold of two occurrences per keyword.

In addition to thematic synthesis, bibliometric analysis was conducted using VOSviewer software (version 1.6.20) to perform keyword co-occurrence analysis of PUFAs in the literature. VOSviewer is a commonly used bibliometric map tool for creating visualisations of the relationships between keywords and, consequently, determining future research directions and lines. At the centre of the analysis was a group of polyunsaturated fatty acids such as GLA, LA, and DGLA, as shown in Figures 1a,b. The labelling of the cluster was based on the frequency and co-occurrence of the corresponding keywords, creating interconnections among the numerous research fields. While VOSviewer enables the visualisation of the scale of relationships, no keyword frequency statistical analysis or trends in cluster emergence were conducted within this study. Full, rather than fractional, counting was employed so that each occurrence of a keyword was counted in its entirety and the landscape of keyword relationships could be defined with a greater degree of granularity, more suitable to the scale of this review.

As compared to fractional counting, this method places equal importance on each instance of a keyword and is not frequency-weighted. This map symbolises the complex web of relationships between GLA, DGLA, and LA, their bioactivities, and their significance in human health. The map also reflected the prime position of these molecules in inflammatory processes, cancer, cardiovascular disorders, and metabolic disorders. Investigating these relationships holds the potential to unveil novel pathways for disease prevention and the development of targeted therapeutic interventions, leading to improved health benefits. Cluster analysis yielded 19 groups, each comprising 8 to 30 keywords, that encompassed different themes related to PUFA studies. The network of keyword co-occurrence positioned GLA, DGLA, and LA at the centre of clusters with connections related to inflammation, obesity, and antioxidants.

Clustering tendency indicated a strong correlation of these fatty acids with inflammatory pathways, as indicated by the dominant orange cluster. GLA and DGLA, as key intermediates of the omega-6 fatty acid pathway, were highly correlated with metabolic and immune responses, indicating their roles in inflammation regulation and lipid metabolism. The blue cluster, rich in antioxidants, is a key area of research emphasis due to its antioxidant activity in preventing oxidative stress induced by lipid peroxidation, which is significantly interconnected with inflammation and obesity. Their interactions with phospholipids and bioactive compounds through LA, GLA, and DGLA also emphasised their role in stabilising cellular membranes and signal transduction. The grouping of the words for phospholipid-related concepts in pink indicates that fatty acids are involved in the function and structure of cell membranes, with the ultimate goal of regulating inflammatory processes. The linking of GLA, DGLA, and LA to the obesity-descriptive words indicates that they are involved in metabolic disturbances and merits further investigation into their use as a drug. Collectively, this visualisation underscores the complex interplay of these fatty acids with basic physiologic processes, affirming their roles in health and disease. The second image is a network visualisation of keyword co-occurrence, where nodes (circles) are terms, and edges (lines) are their co-occurrence in the literature. Larger nodes are more frequent words, and colors mark clusters of relevant terms. At the center of the map are the crucial words such as inflammation, obesity, phospholipids, metabolic syndrome, and chronic diseases, which match their extremely high interconnection with GLA and DGLA.

The blue-to-yellow scale indicates a temporal trend, where blue corresponds to earlier publication dates (2016) and yellow to newer publication dates (2024), and there is an upward trend in interest in subjects such as human cardiotoxicity and neuroprotection. The third image is a density plot illustrating the intensity and frequency of research focus on particular topics. More yellow reflects more research activity, with increasingly blue shadings reflecting relationships with less research. The most researched subjects are GLA, linoleic acid, inflammation, obesity, and phospholipids, which reflect interest in the role that these fatty acids play in inflammation-related diseases and metabolic disorders.

Keywords such as breast cancer, schizophrenia, Alzheimer’s disease, and atopic dermatitis illustrate the development of interest in the therapeutic use of GLA, LA, and DGLA in oncologic, neurological, and inflammatory diseases. The scoping review categorised the current studies into four general themes: the impact of GLA on health, GLA production, manufacturing factors, and directions for future research. These maps were created using VOSviewer (version 1.6.20), employing a complete counting method and a minimum of three mentions per keyword (Figure 1c). A systematic PubMed literature search was conducted to retrieve relevant literature for GLA in biological, chemical, and clinical usage. Literature searching was done with Boolean operators supporting the keywords “gamma-linolenic acid,” “omega-6,” and other similar keywords. Only articles from 2011 to 2024 were chosen, and among these, studies that compared the biochemical, chemical, and clinical significance of GLA were selected. Non-research articles were filtered out to ensure that the dataset consisted only of research studies that report experimental results, therapeutic applications, or biosynthesis pathways of GLA. Out of the total 4,493 documents retrieved from the initial search, these were subsequently screened for inclusion based on specific criteria. Following deduplication and removal of papers unrelated to the research, 1,114 unique studies remained for thematic synthesis. Thematic synthesis revealed trends in research, new themes, and research gaps in the subject area, as well as insights into the evolving understanding of GLA and its potential applications in clinical settings (Figure 1d).

## 3 Sources of GLA

**FIGURE 2:**
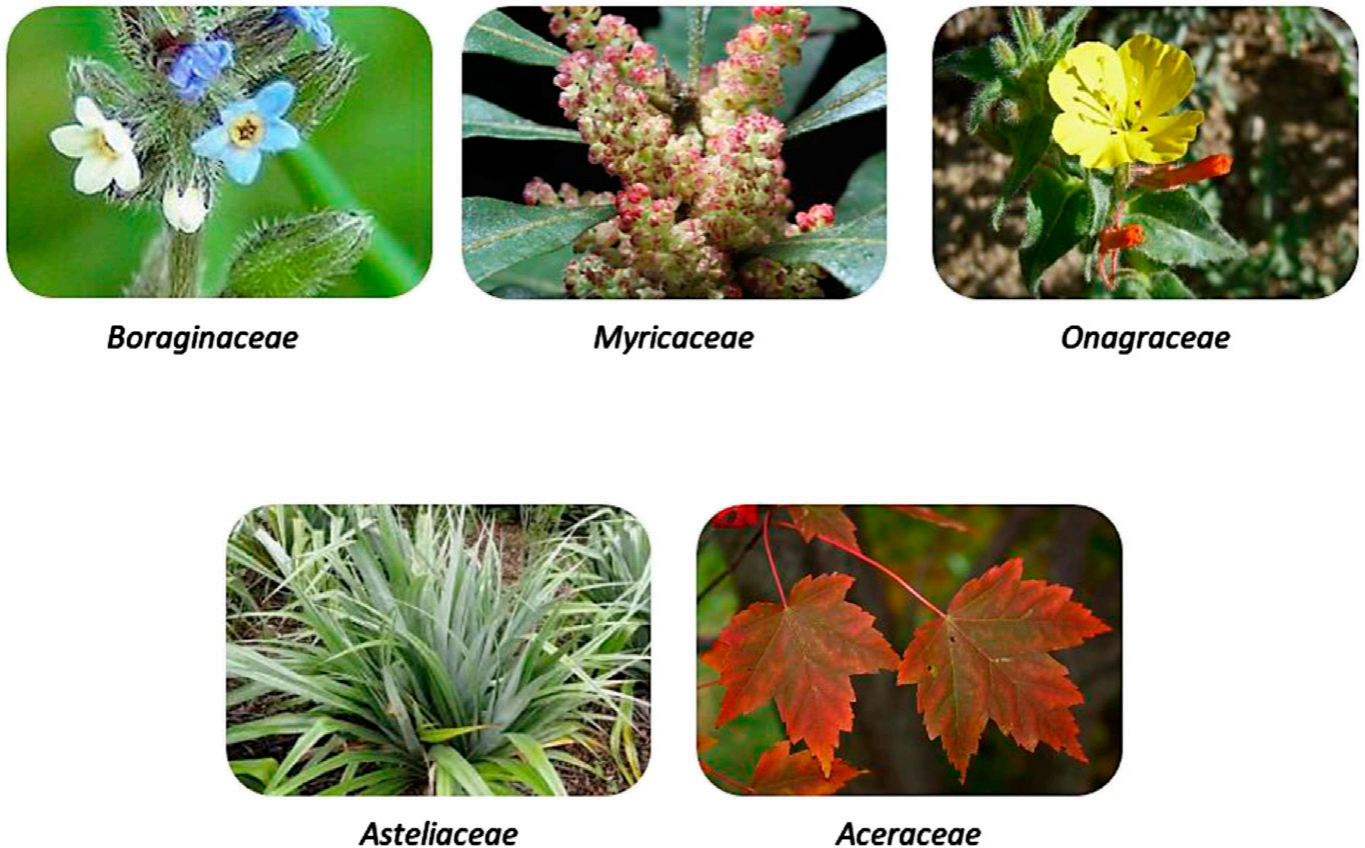
Plant families containing GLA.

Research indicates that GLA is present in various plant families, including Boraginaceae, Myricaceae, Onagraceae, Asteliaceae, and Aceraceae (Table 1; Figure 2). GLA is a molecule synthesised by multiple plants, fungi, yeasts, and algae. However, its chemical synthesis is challenging due to the possibility of isomerisation of its double bonds into *cis* or *trans* configurations, with only the *cis* form naturally occurring. Trans isomers of fatty acids can be less biologically active or even harmful compared to their cis counterparts. GLA is naturally found in the oils of several plant seeds, including borage (*Borago officinalis*), evening primrose (*Oenothera biennis*), and black currant (*Ribes nigrum*). Borage oil contains the highest GLA content, ranging from 20%–27%. Although seeds contain more oil than other parts of the plant, different tissues are also recognised as a source of GLA. The Boraginaceae family is particularly noted for its high GLA content due to the presence of specific enzymes that enhance GLA production. Due to the expense and inefficiency of extracting and purifying GLA from natural sources, there is growing interest in using biotechnological methods for GLA production (Pérez-García and Wendisch, 2018). Biotechnological approaches, such as the metabolic engineering of microbes and various fermentation processes, are being explored to offer higher yields, lower costs, and more sustainable alternatives to plant-based extraction.

**TABLE 1:** GLA-containing organisms.

Kingdom	Family	Organism	GLA%	References
Plantae	Cannabaceae	*Cannabis sativa*	0.5–4.5	Alonso-Esteban et al. (2020)
Plantae	Boraginaceae	*Borago officinalis*	17–25	Kapoor and Nair (2005)
Plantae	Boraginaceae	*Lithospermum purpurocaeruleum*	18	Kapoor and Nair (2005)
Plantae	Boraginaceae	*Onosmodium molle*	20	Kapoor and Nair (2005)
Plantae	Boraginaceae	*Symphaticum officinale*	27	Kapoor and Nair (2005)
Plantae	Grossulariaceae	*Ribes nigrum*	15–20	Ruiz del Castillo et al. (2004)
Plantae	Onagraceae	*Oenothera biennis*	8–14	Engler (1993)
Fungi	Mortierellaceae	*Mortierella vinacea*	22	Amano (1992)
Fungi	Mortierellaceae	*Mortierella ramanniana*	13.2–31.4	Amano (1992)
Fungi	Mucoraceae	*Rhizopus stolonifera* YF6	49	Wan et al. (2011)
Fungi	Mucoraceae	*Rhizopus nigricans* R31.6	24.32	Lu et al. (2009)
Fungi	Mucoraceae	*Rhizopus oryzae*	5.6–15.3	Kawashima et al. (2002)
Fungi	Mortierellaceae	*Mortierella isabellina*	20.2	Streďanská and Šajbidor (1992)
Fungi	Mucoraceae	*Mucor circinelloides*	28.6	Streďanská and Šajbidor (1992)
Fungi	Cunninghamellaceae	*Cunninghamella elegans*	13.5	Streďanská and Šajbidor (1992)
Bacteria	Microcoleaceae	*Spirulina platensis*	18–21	Mendes et al. (2006)

## 4 Biosynthesis and bioengineering

### 4.1 GLA biosynthetic pathway

**FIGURE 3:**
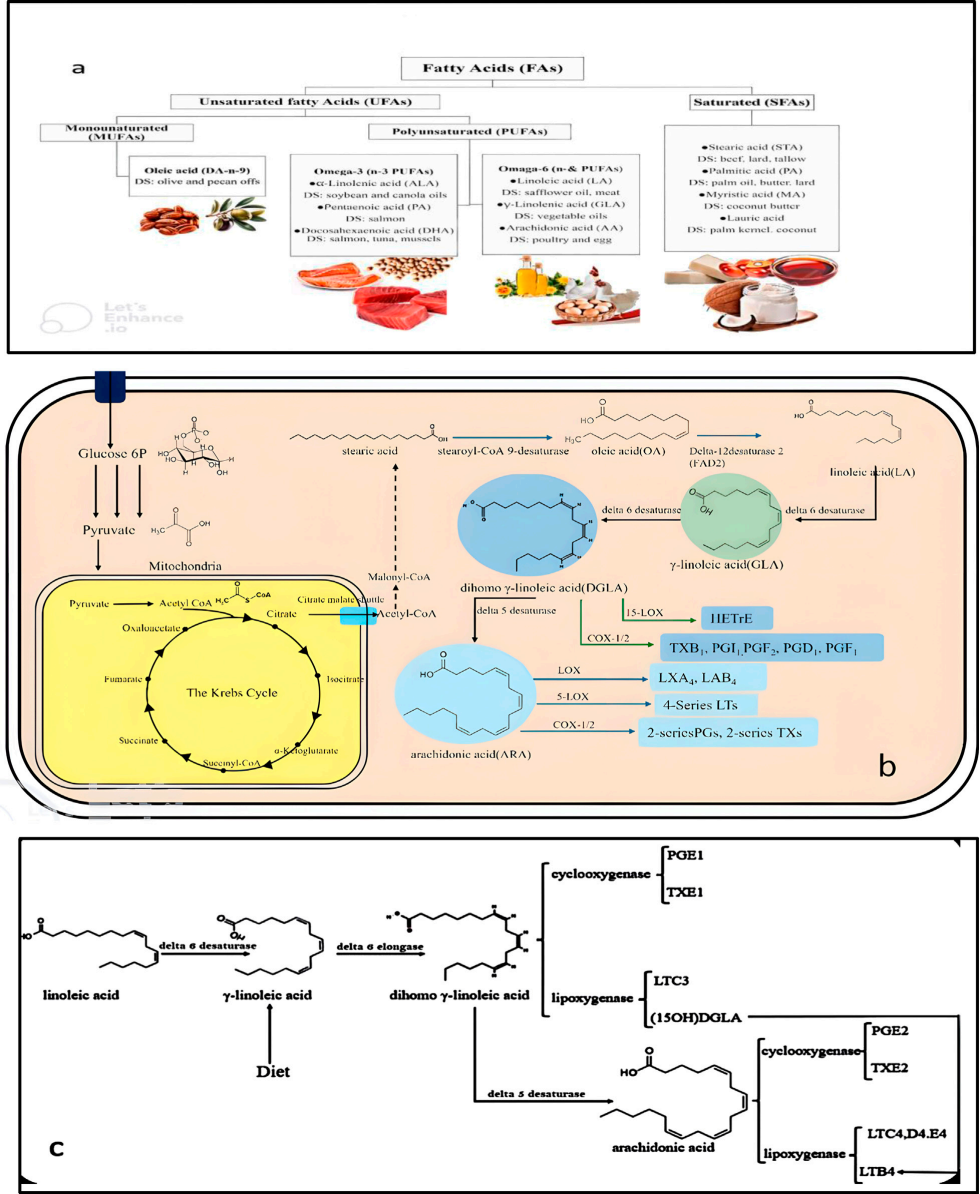
Classification of fatty acids, sources, biosynthesis, and GLA. **(a)** Saturated (SFAs) and unsaturated (UFAs) are the two categories of fatty acids. Among the unsaturated fatty acids, there are monounsaturated fatty acids (MUFAs) and polyunsaturated fatty acids (PUFAs). Gamma-linolenic acid (GLA) is a polyunsaturated fatty acid (PUFA). DS: Dietary sources. **(b)** Fatty acid biosynthetic pathway: The biosynthesis of fatty acids in eukaryotic cells generally follows a common scheme initiated with acetyl-CoA and malonyl-CoA, culminating in the formation of fatty acids such as palmitic and stearic acids. Oleic acid (OA), an omega-3 and omega-6 pathway precursor, is formed, and the reaction is initiated when glucose is transported into the cell through specific glucose transporter proteins. The glucose is phosphorylated to glucose-6-phosphate, which is catabolised via glycolysis to yield pyruvate. Pyruvate is fed into the mitochondria and reduced to acetyl-CoA by the complex of pyruvate dehydrogenase. Acetyl-CoA passes into the Krebs cycle and is converted into intermediates like citrate, succinyl-CoA, and malate, before getting converted back to Acetyl-CoA in the cytoplasm. This Acetyl-CoA is carboxylated into malonyl-CoA, which is extended into chains to form stearoyl-CoA, and desaturated into OA. OA is then desaturated to linoleic acid (LA), which is further desaturated to GLA, DGLA, and finally ARA. ARA is metabolised to leukotrienes (LTs), lipoxins (LXs), and cyclooxygenase products such as 2-series prostaglandins (PGs) and thromboxanes (TXs). DGLA is also metabolised via various pathways to yield hydroxy eicosatrienoic acids (HETEs) and other eicosanoids such as TXB1, PGI_1_, PGF_2_, PGD_1_, and PGF_1_. **(c)** GLA is converted to DGLA in the liver, and DGLA can be metabolised in three ways: it can be reduced to prostaglandin PGE_1_ by COX, to 15-hydroxy eicosatrienoic acid (15-HETrE) by lipoxygenase, or to ARA by D5D. The anti-inflammatory activity of DGLA is obtained in three ways: it replaces ARA in inflammatory cells’ membrane, blocks the metabolism of ARA by COX and lipoxygenase by competing with the latter, and forms anti-inflammatory PGE_1_ that dilates pulmonary vessels. DGLA also forms 15-HETrE and blocks the formation of LTB_4_ by ARA and is anti-inflammatory.

Omega-6 fatty acids, a class of polyunsaturated fatty acids (PUFAs) (Figure 3a), including LA, ARA, GLA, and DGLA, which are primarily found in eggs, nuts, seeds, vegetables, corn oil, and sunflower oil (Czumaj and Śledziński, 2020). These fatty acids are synthesised by the enzymatic conversion of Acetyl-CoA derived from carbohydrates via the glycolytic pathway and NADPH, generated by the malic enzyme in the pentose phosphate pathway, in the cytoplasm. However, the specific pathways for GLA synthesis differ among the organisms producing it. In humans and other animals, GLA is formed following the desaturation of LA as the first step in the synthesis of ARA (Figure 3b) (Kapoor and Huang, 2006; Hernandez, 2016; Simopoulos, 2021). In contrast, this process may differ in microorganisms and plants, reflecting the diversity of enzymatic pathways in various species.

Functionally, GLA is a precursor to prostaglandins and leukotrienes, which are involved in inflammation and immune responses. The enzyme delta-6 desaturase (D6D) catalyses the desaturation of LA into GLA, and delta-6 elongase (D6E) converts GLA into DGLA. This DGLA subsequently modulates the biosynthesis of various eicosanoids, such as prostaglandins (PGs), leukotrienes (LTs), thromboxanes (TXs), and lipoxins, via cyclooxygenase (COX) and lipoxygenase (LOX) pathways (Kapoor and Huang, 2006). Specific derivatives like PGD_2_, PGE_2_, PGI_2_, PGH_3_, and LTB_4_ play vital roles in mediating inflammation, vasodilation, platelet aggregation, and immune signalling (Figure 3c). Importantly, anti-inflammatory mediators such as prostaglandin E_1_ (PGE_1_) and 15-HETrE, originating from GLA metabolism, are crucial in regulating fever, smooth muscle contraction, and immune modulation. GLA also influences COX and LOX activity by promoting the synthesis of anti-inflammatory molecules, thereby contributing to immune regulation (Peng et al., 2022; Jang and Park, 2020; Lin et al., 2013; Barry and Dixon, 2021; Gammone et al., 2019; Meechai et al., 2004; Novichkova et al., 2020; Mustonen and Nieminen, 2023; Ikeh et al., 2018; Thurairatnam, 2012). In response to bacterial infections, eicosanoids such as PGE_2_, PGA_2_, and series 4 leukotrienes exhibit antibacterial properties and balance inflammatory processes.

Prostaglandins, which are regulated by fatty acids, play essential roles in various bodily functions, including the regulation of organs and immune responses, and contribute to the pathophysiology of cancer and inflammation. Cyclooxygenase enzymes (COX) convert omega-6 fatty acids into prostaglandins, which, in turn, influence cancer cell proliferation. COX inhibitors, such as nonsteroidal anti-inflammatory drugs (NSAIDs), help reduce inflammation and pain (Zarghi and Arfaei, 2011). Gamma-linolenic acid demonstrates promising potential in cancer therapy by selectively inducing apoptosis in cancer cells, while sparing normal cells. It has been shown to disrupt the cancer cell cycle and promote cell death. Moreover, GLA may help prevent inflammatory diseases by converting into dihomo-DGLA, which produces powerful anti-inflammatory agents. Given its anti-inflammatory and immune-modulating effects, GLA holds promise as an adjunct in cancer therapy, although its selective anticancer properties require further investigation (Shi et al., 2023).

### 4.2 Genetic engineering in GLA-producing organisms

One of the purposes of genetic engineering is to enhance the product yield and efficiency in various systems. Genetic engineering has been utilised in organisms such as *Yarrowia lipolytica* and *Mucor circinelloides* to enhance GLA production. In *Yarrowia lipolytica*, the directed integration of genes encoding delta-12 and delta-6 desaturases increased production. In *M. circinelloides*, directed evolution techniques were employed to engineer variants with enhanced stability and activity for the treatment of Fabry disease. In plant systems, attempts have been made to produce fatty acids within chloroplasts, but the efficiency and stability of these approaches are still being investigated (Salah et al., 2019).

The selection of host organisms for GLA production must consider multiple factors. The challenges in genetic engineering for production include optimising the expression of critical enzymes involved in the biosynthetic pathway, enhancing production efficiency in host organisms, and ensuring the stability and scalability of production processes (Wu C. et al., 2023). Additionally, challenges may arise in maintaining the integrity of genetic modifications over successive generations of organisms, addressing potential regulatory hurdles related to genetically modified organisms, and optimising fermentation conditions to maximise yield while minimising production costs (Mirbagheri Firoozabad and Akhbariyoon, 2022). These challenges require innovative genetic engineering strategies, robust metabolic engineering approaches, and a thorough understanding of the biochemical pathways involved in GLA biosynthesis to overcome obstacles and achieve efficient and sustainable production. In GLA biosynthesis, three desaturases, delta-9, delta-12, and delta-6, are involved. The genes encoding these desaturases have been cloned from existing prokaryotes to advanced eukaryotes and overexpressed in varieties of hosts, including microalgae, yeast, and plants.

This section examines various host organisms, including plants, fungi, yeasts, algae, and moss, utilised in genetic engineering to enhance GLA production. Each subsection presents valuable insights into the genetic strategies implemented across different systems. The text is rich in detail, offering thorough descriptions of genetic engineering approaches, such as the overexpression of desaturases and gene deletions, which have been shown to improve GLA production in yeasts and fungi. Additionally, comparing the effectiveness of these genetic modifications across various host systems, such as plant-based, microbial, and fungal systems, in terms of yield, scalability, and sustainability would provide a more nuanced perspective.

#### 4.2.1 GLA engineering of plants and mosses

Plants offer advantages such as genetic manipulability, suitability for large-scale cultivation, and are generally regarded as safe (GRAS). However, plants may produce GLA at different levels than other organisms, making them less efficient for production. Currently, GLA sources are limited to a few plant species with weak agronomic properties. Therefore, developing a commercially viable and economical source could be desirable. One approach has been to transfer the *D6D* gene to convert LA to GLA in oilseed plants (Zhang et al., 2017). Table 2 summarises some plants used as hosts for GLA production (Wu K. et al., 2023). The advantages of plant-based GLA production include cost-effectiveness, compared with traditional chemical synthesis methods, being inherently a renewable resource with sustainable growth, with minimal environmental impact, and efficient in carbon sequestration. Additionally, plant-based GLA can offer nutritional benefits, including omega-3 fatty acids. However, there are challenges and drawbacks, such as limited sources, with only a few plant species currently able to produce significant amounts of GLA, agronomic limitations, as many plants with high GLA content have limited agronomic properties, making large-scale cultivation challenging, and the need for genetic modification, which is often required to enhance GLA production in plants, and can be costly and time-consuming. Recent advancements, such as CRISPR-Cas9 (Kan et al., 2022), have enabled more efficient and targeted genetic modification, leading to the development of crops with enhanced nutritional value, disease resistance, and stress tolerance. These technologies have improved the precision and efficiency of plant genetic modification and have been successfully applied in GLA production. For instance, CRISPR-Cas9 has been used to create plant variants with enhanced GLA content, demonstrating the potential for genetic engineering to optimise GLA yield. In the future, there is likely to be more research focused on optimising plant-based GLA production through genetic engineering and other technologies. Highlighting the potential for developing new plant species or hybrids that could improve GLA yield further reinforces the need for biotechnological advances (Kan et al., 2022).

**TABLE 2:** Genetic engineering of plants to increase GLA production.

Organism	Gene	Gene source	Vector	Promoter	Culture medium	Changes in GLA content	References
*Brassica napus* ^*1*^	*D6D*	*Phytophthora citrophthora* KACC 40188	pCAMBIA-PcD6DES used as a binary vector	vicilin	liquid MS + (BA) + (NAA)	more than 25% in T2	Lee et al. (2019)
*Carthamus tinctoriu* ^*2*^	*D6D* *&D12D*	*Mortierella alpina*			Explants were incubated on media for co-cultivation and then transferred to media containing vimentin to inhibit Agrobacterium growth. Subsequently, explants were selected on media containing glufosinate-ammonium	50%	Nykiforuk et al. (2012)
*Carthamus tinctorius (Safflower)* ^*2*^	*D6D*	*Saprolegnia diclina*	plant binary expression vectors pSBS4119, pSBS4763 and pSBS4766	ubiquitin promoter/terminator		73%	Nykiforuk et al. (2012)
*Nicotiana benthamiana* ^*3*^	*D6D* *&D6E* *&D5D*	*Micromonas pusilla* *D6D* *Pyramimonas cordata* *D6E* *Pavlova salina* *D5D*	pWVEC8	CaMV35S linin (seed-specific *Linum usitatissimum*)		2.1%	Petrie et al. (2010)
*Nicotiana benthamiana* ^*3*^	*D6D* *&D6E* *&D5D*	*Echium plantagineum* *D6D* *Pyramimonas cordata* *D6E* *Pavlova salina* *D5D*	pWVEC8	CaMV 35S		4.4%	Petrie et al. (2010)
*Nicotiana benthamiana* ^*3*^	*D6D* *&D6E* *&D5D*	*Ostreococcus tauri* *D6D* *Pyramimonas cordata D6E* *Pavlova salina* *D5D*	pWVEC8	CaMV 35S		5.8%	Petrie et al. (2010)
*Glycine max (L.)* *Soybean* ^*4*^	*D6D* *&* *D15D*	*Pyramiding of borage D6D & Arabidopsis D15D*	two T-DNA binary plasmids, pPTN382	seed-specific soybean betaconglycinin promoter	Transformants were selected on glufosinate at 5 mg/L during shoot initiation and 3 mg/L during shoot elongation. Primary transformants were grown to maturity under greenhouse conditions	Up to 31%	Eckert et al. (2006)
*Brassica juncea* ^*5*^	*D6D* *&D6E* *& D5D* *& D2D*	*D6D P. Irregular* *D5D Thraustochytrium* sp. *26185* *D6E patens* *D12D C. officinalis* (Gateway system)		napin promoters		27–29 %	Eckert et al. (2006)
*Arabidopsis thaliana* ^*6*^	*D5D* *&D6D* *&D6E* *&D5E* *&D4D*	*Danio rerio* *D5D/D6D, Caenorhabditis elegans* *D6E* *Pavlova salina* *D5E* *P. salina* *D4D*	pENTRY	Cnl USP SBP1800	T1 plants were established from seedlings recovered following germination on combined hygromycin and kanamycin media	0.4%	Robert et al. (2005)
*Oenothera biennis* ^*7*^	*D6D*	*Borago officinalis L*	plasmid pNTdes6	CaMV35S	5 MS media +TDZ IBA	8%–21% in leaves	Robert et al. (2005)
*Glycine max (L.)* ^*8*^	*D6D*	*Borago officinalis L*	Two T-DNA Binary Vector pPTN331	embryo-specific promoter beta-conglycin		up to 28.7%	Sato et al. (2004)
*Nicotiana tabacum* *&* *Linum usitatissimum* ^*9*^	*D6D* *&* *D6E* *&* *D5D*	*PpD6, BoD6,&* *PtD6 from P.patens (AJ222980),* *B. officinalis (U79010), &* *P.tricornutum (AY082393)* *MaD5 &PtD5, from M.alpina (AF054824)& P.tricornutum (AY082392),* *PSE1 &PEA-1, from P.patens (AF428243)& C. elegans(F56H11.4)*	binary vectors pCAMBIA2300 & pGPTV	Seed-specific promoters CaMV35S & USP promoter (region of the unknown seed protein of *Vicia faba*) &LeB4 promoter (of the legumin gene of V. faba) Dc3, promoter (of the helianthinin gene of *Daucus carota*)	MS medium	30% in tobacco & 17% in Linum	Abbadi et al. (2004)
*Brassica napus*	*D6D* *& D12D* *&D15D*	*pyramiding of D6D* *& D12D* *M. alpina &* *D15D B. napus*					Ursin (2003)
*Brassica junca* ^*10*^	*D6D* (*PiD6*)	*Pythium irregular* *10,951*	TA cloning vector	*Brassica napus* napin promoter	liquid medium consisting of 3 g/L yeast (*Saccharomyces cerevisiae*) extract, 3 g/L malt extract, 5 g/L peptone, 10 g/L Glc, 0.68 g/L K_2_HPO_4_, pH 6.0, and 1 n HCl	25–40 % (Up to 40% in seeds)	

Transformation method:1: *A. tumefaciens* EHA105, 2: *Agrobacterium,* 3: *A. tumefaciens,* 4: *A. tumefaciens* strain EHA101, 5: *A. tumefaciens* GV3101 (pMP90), 6: floral dip (Gateway recombination system), 7: *A. tumefaciens* hypervirulent strain AGL1, 8: *A. tumefaciens* strain EHA101, 9: *A. tumefaciens* strain C58C1 ATHV, 10: *A. tumefaciens* GV3130: pMP90.

In this context, mosses have emerged as an alternative plant-based platform, particularly in the model organism *P. patens*, has been significantly advanced through genetic engineering (Beike et al., 2014). Researchers have successfully altered fatty acid biosynthetic pathways by leveraging extensive knowledge of plant oil biosynthesis and employing genetic tools. For instance, the heterologous expression of a *D5E* gene from the algae *Pavlova* sp. under the control of a tandemly duplicated 35S promoter enabled the production of ADA in *P. patens*. This gene facilitated the conversion of endogenous ARA to ADA, achieving a production level of 0.42 mg/L, which was further optimised to 4.51 mg/L using RSM and increased GLA and DGLA content. This study marks a pioneering effort in expressing a PUFA-synthesising enzyme in a non-seed, lower plant without the need for exogenous fatty acid supplementation (Kaewsuwan et al., 2010; Chodok et al., 2012). Moss-based systems, such as *P. patens,* offer a unique alternative to traditional plant-based systems for GLA production. Unlike seed plants, mosses can be grown in controlled environments without large agricultural spaces. Mosses can be genetically engineered to produce high levels of GLA and other valuable compounds, making them a promising option for researchers exploring alternative non-seed plant hosts for biotechnological applications.

#### 4.2.2 GLA engineering of fungi and yeasts

Fungal genetic engineering can increase GLA production by improving yield, efficiency, and cost-effectiveness compared to traditional chemical synthesis. However, challenges such as scalability issues, high production costs, and potential negative impacts on fungal growth and viability must be addressed. To overcome these challenges, it is essential to optimise genetic engineering techniques, select suitable fungal strains, develop efficient fermentation processes, and explore cost-reduction strategies. The potential of fungal-based GLA production can be effectively realised by addressing these factors.

• Methods to enhance DGLA production include overexpressing the transketolase gene and disrupting a glucan synthase gene.
• Researchers have achieved a 1.9-fold increase in free DGLA yield to 403 mg/L by overexpressing an anticipated transketolase gene in DGLA3.
• Disturbing the α-1,3-glucan synthase gene *agsB*, involved in cell-wall biosynthesis, has increased the content by 1.3-fold–533 mg/L, resulting in a 2.5% increase in overall yield.

These findings are illustrated in Table 3, which depicts the genetic engineering of fungi to increase the amount of GLA (Tamano et al., 2023).

**TABLE 3:** Genetic engineering of fungi to increase the amount of GLA.

Organism	Strain	Gene	Gene info	Gene source	Vector	Promoter	Transformation method	Culture medium	Cultivation condition	GLA content	References
*Aspergillus oryzae*	BCC 14,614 GLA = ND	*D6D* */D6E* *& mMaDGAT2*	codon-opti complet CDS (MK091394)	*Pythium sp* *&* *M. alpina*	pAMDes6-MElo6	Enolase (Penol)	PEG-mediated	production media	high C: N culture (29:0; 6% glucose) 72 h	198 mg/L	Jeennor et al. (2019)
*Mucor circinelloides*	CBS 277.49	*D6D 1,2* *&* *D12D*	CDS	*Mucor circinelloides* *CBS 277.49*	pMAT1552 (pMD19T-CarRP)	zrt1	electroporation	K&R media	500 mL Flasks+ 100 mL K&R 24 h 30°C/150 rpm inoculated at 10% in 2 L fermenter +1.5 L K&R +80 g glucose/L 500 rpm aeration 0.5 vvm. pH = 4.5	43% or 180 mg/L 33% increase	Jeennor et al. (2019)

The *D6D* gene from *Mucor rouxii*, involved in GLA formation, was introduced into *Hansenula polymorpha* yeast. Changes in essential fatty acid synthesis pathways were observed, with GLA production varying based on growth conditions (Laoteng et al., 2005). One study on 28 Zygomycetes fungi revealed that strains exhibiting low oleic acid synthesis due to reduced delta-9 desaturase activity displayed maximal delta-15 desaturase activity. Delta-6 desaturase activities indicated competition among fatty acids in n3, n6, and n9 biosynthetic pathways. Understanding these fatty acid desaturase activities presents new opportunities for optimising the biotechnological production of PUFAs by Zygomycetes fungi (Klempova et al., 2013).

The ease of genetic manipulation and the high efficiency of homologous recombination in yeasts make them ideal platforms for synthetic biology projects and industrial biotechnology applications (Table 4) (Schindler, 2020; Taghon and Strychalski, 2023; Gupta et al., 2024). The codon*-*optimised *D6D* from *M. alpina* was introduced into *Yarrowia lipolytic,* controlled by the solid hp4d promoter. The engineered strains produced a total fatty acid profile containing 4.6% GLA (Gu et al., 2023).

• In a study on two strains of *Y. lipolytica* (strain Po1f-Δk and 2Pg2E), researchers produced GLA by eliminating specific genes. They removed the *KU70* gene from strain Po1f to create Po1f-Δk and the *FAD2* gene to produce strain 2Pg2E. During shake flask fermentation, the synthesised strains produced GLA, which accounted for 22.58% of the total fatty acids (TFAs). Additionally, the corresponding total titers reached 386.59 mg/L.
• Scientists are focusing on modifying the endogenous pathways of yeast, particularly *Y. lipolytica,* by expressing critical enzymes involved in GLA biosynthesis, such as Acetyl-CoA synthase, Acetyl-CoA carboxylase, and fatty acid synthesis enzymes. These modifications improve the yeast’s ability to utilise acetate during fermentation, increasing lipid production and GLA content (Mirbagheri Firoozabad and Akhbariyoon, 2022).
• Strategies such as inhibiting β-oxidation through genetic deletions and editing the diacylglycerol biosynthesis pathway have been employed to enhance GLA production in yeast cultures further. By optimising these genetic and fermentation processes, researchers are working towards developing sustainable and efficient methods for mass-producing in engineered oleaginous yeasts like *Y. lipolytica* to meet the increasing demand for this essential fatty acid in various applications (Firoozabad and Akhbariyoon, 2021).

**TABLE 4:** Genetic engineering of yeasts to increase GLA production.

Organism	Strain	Gene	Gene info	Gene source	Vector	Promoter	Transformation method	Culture	Fermentation condition	GLA content	References
*Y. lipolytica*	Po1f GLA = ND	*D6D* *& D12D*	codon-optimized *FAD* genes	*Mortierella alpina*	vector p0 + URA3 selection marker	hp4d	lithium acetate method	YPD medium	shake flask fermentation (28°C 1day & culture at 20°C for 6 days)	71/6 mg/L 4.6% transgene 60.9% Increase in temp strategy	Sun et al. (2017)
*Saccharomyces cerevisiae*	INVScl	*D6D*	full-length cDNA	*Rhizopus oryzae* DR3	pYRoD6D		lithium acetate method	Douchi		14/24%	Lu and Zhu (2015)
*Saccharomyces cerevisiae*	INVScl	*D6D* AY795076	full-length cDNA	*Rhizopus stolonifer*	pLYRsD6D pSYRsD6	GLA1	lithium acetate method	synthetic minimal medium (SC–Ura)		6/23% in pLYRsD6D 1/84% in pSYRsD6	Lu and Zhu (2015)
*Hansenula polymorpha*	KYC625 methylotrophic	*D6D*		*Mucor rouxii*		MOX	lithium acetate method	GBS glycerol-limited conditions	Fed-Batch Fermentation 28% of DOT, 1 g/L yeast extract and 3.6 mL/L PTM1	10%	Khongto et al. (2011)
*Hansenula polymorpha*	KYC625	*D6D*		*Mucor rouxii*	pMG281	methanol oxidase (MOX)	lithium acetate method	glycerol basal salt (GBS)	Fed-Batch Fermentation 30°C, aeration 1–4 L/min, pH 5	697 mg/L	Khongto et al. (2010)
*Pichia pastoris*	GS115 GLA = 0	*D6D*	complete cds (DQ291156)	*Rhizopus stolonifera* strain YF6	pHBM954	AOX1	electroporation	BMGY methanol as C	16–18 h in 72 h at 20°C	22/4%	Wan et al. (2009)
*Saccharomyces cerevisiae*	W303-a	*D6D*	full-length cDNA	*O. biennis*	pYES2-ObD6D		lithium acetate method		galactose (2%, w/v) for 48 h at 20°C	3/1%	Huang et al. (2010)
*Pichia pastoris*	GS115	*D6D*	Full-length cDNA	*Cuninghamella echinulata* strain MAIN6	pHBM605	AOX1	electroporation	BMGY m.	72 h at 20°C	1/3%	Wan et al. (2009)
*Saccharomyces cerevisiae*	INVScl	*D6D*	full-length cDNA	R. nigricans *R31.6*	pYRnD6D	GLA1	lithium acetate method	synthetic minimal medium (SC–Ura)	2.5% galactose 30°C &15°C	6.25% & 13.69% at 30°C 22.23% at 15°C	Lu et al. (2009)
*Saccharomyces cerevisiae*	INVScl	*D6D*		*R. stolonifer* strain As3.3						12/25%	Zhang et al. (2004)
*Saccharomyces cerevisiae*	SC334	*D6D* *& D12D*		*M.alpina*		GAL1 & TPI1				8%	Mingchun et al. (2003)

Recently, a study was conducted to improve the expression of *D6D*, a necessary enzyme in the GLA pathway. For the clone *M. rouxii* delta-6 desaturase, the expression vector pPICZC was used. In *Escherichia coli*, the engineered vector was first cloned. *E*. c*oli* DH5α and, after plasmid extraction and sequencing confirmation, was transformed by electroporation into *Pichia pastoris GS115*. The results showed that in the presence of 0.5% methanol, a genetically modified yeast strain expresses this gene. Lipids and essential fatty acids, particularly GLA, have been obtained to verify the expression. In the studies on the production of lipids and fats, a recombinant strain generated GLA levels up to 19.2% of overall fat content by Sudan black and Nile Red staining, GC, and flow cytology (Mirbagheri Firoozabad and Akhbariyoon, 2022; Chaudhary et al., 2024). The yeast section could benefit from more context regarding the industrial relevance of these modifications. For instance, genetically engineered yeasts provide advantages in terms of scalability and production cost compared to other hosts, making them a promising option for the commercial production of GLA.

Oleaginous yeast technology offers an attractive means of augmenting the yield of lipids and GLA without the challenges of using microalgal sources. Microalgae-derived GDGs and GLA are structurally complex, rare, and difficult to isolate or produce, even with significant demand. *Y. lipolytica* is a potential alternative, as it is capable of metabolising hydrophobic substrates and has the potential to redirect its metabolic pathway capabilities toward lipid biosynthesis. Its robust lipid accumulation routes and stress tolerance qualify it to be an organism for the large-scale production of GLA. Lipid biosynthesis in *Y. lipolytica* has been advanced significantly by synthetic biology, gene editing, and metabolic engineering. Advances in biosynthetic genes, metabolic pathways, and fine-tuning of regulation have contributed to enhanced GLA productivity. Moreover, strategies such as adaptive evolution, cocultivation, and bioprocessing have also been optimised for lipid accumulation. Engineering *Y*. *lipolytica* for GLA includes redesigning glycosylation pathways and fatty acid biosynthesis to achieve stereoselective synthesis. Subsequent studies will emphasize integrating omics technologies, optimisation of substrate metabolism, and yeast strain engineering towards achieving high-yield, low-cost, and environmentally friendly biosynthesis of GLA (Gu et al., 2023).

Manipulation of *Y. lipolytica*, both in its genetic background and in culture conditions, has resulted in successful improvement of lipid storage as well as elevating the ω-6 PUFA biosynthesis involving GLA and DGLA. Scientists have optimized the Δ6 and Δ8 biosynthetic pathways of *Y. lipolytica*, which were initially sourced from *M. alpina* and *Isochrysis galbana*, respectively. The Δ6 pathway is more effective for GLA production, while the Δ8 pathway is more effective for the production of DGLA and ARA. Genetic engineering technologies such as the deletion of *KU70* to increase homologous recombination and *FAD2* to regulate fatty acid desaturation have significantly influenced lipid composition. Additionally, metabolic engineering technologies with strategies to increase precursor supply and suppress substrate competition have also improved ω-6 PUFA production. As a result, engineered strains have exhibited high lipid productivity, with DGLA reaching up to 46.65% of TFAs, confirming the viability of *Y. lipolytica* as an effective microbial platform in producing industrial ω-6 PUFAs. Optimisation of culture conditions has also played a critical role in favouring lipid and ω-6 PUFA accumulation in genetically modified *Y. lipolytica* strains. For example, Po1f-Δk and 2Pg2E strains were developed to increase homologous recombination efficiency and initiate OA production, respectively. Genetic engineering led to significant lipid composition alteration, where Po1f-Δk contained 37.52% OA and 8.32% LA of TFAs, and 2Pg2E yielded an OA ratio of 82.31%. The lipid content was 8.50%–14.55% DCW, an indication of the success of selective genetic improvement. Additional optimisations of ω-6 PUFA titers were realised through the alleviation of substrate competition and optimisation of necessary biosynthetic precursors (Wang et al., 2023).

#### 4.2.3 GLA engineering in algae

Algae genetic engineering holds promise for biofuel production, carbon capture, and the synthesis of valuable compounds such as omega-3 fatty acids and pigments (Kumar et al., 2020). Advances in genetic tools and techniques enable the modification of algae to enhance photosynthetic efficiency, increase biomass yield, and produce target metabolites, thereby contributing to sustainable energy solutions and bioproducts (Grama et al., 2022). Algae have been found to contain the necessary genes for GLA production, including the enzymes D6D and D5D. For example, the green algae *Chlamydomonas reinhardtii* has been discovered to have a D6D enzyme capable of converting LA into GLA. Researchers have tried to modify the genetic makeup of algae to produce GLA. A study successfully introduced a *D6D* gene from *M. alpina* into *C. reinhardtii*, leading to increased GLA production. Another study engineered the green algae *Scenedesmus obliquus* to produce GLA by overexpressing a *D6D* gene from *Arabidopsis thaliana*. Additionally, synthetic biology methods have been explored to create new pathways for GLA production in algae (Campos et al., 2020). Microalgae face several challenges in their use for GLA production. These include slow growth rates, lower GLA yields compared to heterotrophic hosts, high infrastructure, operation, and maintenance costs, as well as difficulties in engineering native host microalgae for GDGs-GLA production. Additionally, seasonality and geographical location can limit algae availability and output, while the lack of regulation for algae as dietary supplements poses further challenges (Bleakley and Hayes, 2017). Algae can be genetically engineered to enhance photosynthetic efficiency, potentially leading to higher biomass yield and target metabolite production. Algae are also capable of carbon capture, contributing to sustainable energy solutions. Like yeast or fungi, algae often face slow growth rates and lower GLA yields. The high infrastructure and operation costs and environmental dependencies, such as seasonality and geographical locatio,n add to the complexity of using algae for GLA production.

### 4.3 Culture condition engineering

The culture conditions in engineering play a crucial role in enhancing the production of lipids and GLA in microorganisms. Studies on *Mucor rouxii* and *Mucor* sp.1b have provided valuable insights into the optimal culture conditions for high accumulation. These strains thrive at low temperatures, around 25°C, and with vigorous light intensity, specifically at 6 kLux. Additionally, adding a primrose oil supplement at a concentration of 0.8% w/v has further enhanced production in these strains. The choice of carbon and nitrogen sources significantly affects lipid and GLA production. Specific carbon sources, such as glucose, sucrose, starch, and lactose, have been identified as influential factors in the growth and metabolism of these microorganisms. Similarly, nitrogen sources like potassium nitrate have been found to impact lipid and GLA production by carefully selecting and optimising these nutrient sources. Scientists can create a favourable environment for the organism, increasing lipid and GLA production. Culture condition engineering is a multifaceted approach that involves manipulating specific cultural parameters to create a favourable environment for the organism to boost GLA production. This can include adjusting temperature, light intensity, and nutrient sources and employing other genetic and metabolic engineering techniques (Table 5) (Hansson and Dostálek, 1988; Certik et al., 2012).

**TABLE 5:** Effective parameters on culture optimisation.

Parameter	Role	References
Carbon source	The choice of carbon source affects the overall lipid and GLA production in microorganisms. Carbon sources, such as glucose, sucrose, starch, and lactose, influence lipid and GLA production in fungi	Certik et al. (2012)
Nitrogen source	Nitrogen sources also impact GLA production. A high carbon-to-nitrogen (C/N) ratio in the medium leads to increased lipid and GLA production in particular fungi, such as *Mucor circinelloides* WJ11, which produces 36% lipid of its cell dry weight when cultured in a high C/N ratio medium	Certik et al. (2012)
Mineral salts	Mineral salts in the medium contribute to the organism’s overall health and growth, thereby influencing the production of GLA. An optimised level of mineral salts is required for efficient GLA synthesis	Ronda et al. (2014)
pH	The pH of the medium has a significant impact on the growth and metabolism of microorganisms, including GLA production. Optimal pH levels for GLA production have been identified for various fungi and cyanobacteria	Ronda et al. (2014), Shivaramu et al. (2008)
Other components	Other components in the medium, such as trace elements, vitamins, and growth factors, can also influence GLA production. Their inclusion in the medium needs to be carefully balanced to ensure optimal growth and GLA synthesis	Ronda et al. (2014), Shivaramu et al. (2008)

Note: In wild-type Zygomycetes grown under lipid-accumulating conditions, GLA, content is generally low. Higher GLA, percentages are observed under nitrogen-rich or non-lipid-accumulating.

Optimising culture conditions, such as temperature and carbon source,s has enhanced GLA production in fungi. The two methods are solid-state fermentation (SSF) and submerged fermentation (SMF). SSF is superior due to its lower water activity, which supports better growth and metabolite production in some fungi.

#### 4.3.1 Culture optimization in plants and mosses

The production of GLA in plants is significantly affected by salinity stress (Urrestarazu et al., 2018). Salinity increases GLA production by activating specific stress-induced pathways that benefit GLA biosynthesis. These pathways enhance the plant’s ability to produce and accumulate GLA in response to stress conditions. Researchers have investigated the impact of salinity on GLA accumulation, discovering that various factors, including light intensity and quality, temperature, carbon dioxide concentration, nutrient availability, water stress, and plant hormones, influence GLA production. For instance, increasing light intensity and using blue or red light can enhance GLA production more effectively than white light. The optimal temperature range for GLA production is typically between 18°C and 22°C, and elevated CO_2_ concentrations can increase photosynthesis and, consequently, GLA production. Nutrient deficiencies, especially nitrogen and phosphorus, can limit GLA production. At the same time, water stress and plant hormones can affect gene expression and metabolic pathways involved in fatty acid synthesis, thus impacting GLA production. Researchers aim to enhance GLA production in plants for industrial applications by optimising culture conditions through genetic engineering, environmental manipulation, and bioreactor design. Studies have also indicated increased oil content and specific fatty acid compounds, like PA, SA, and GLA, at higher salinity levels. Moreover, a positive correlation exists between *D6D* gene expression and GLA content, particularly at high salinity levels. Mit Wan utilised saline water as an alternative source for *Oenothera biennis* production in plant cell culture (Shahbazi et al., 2018).

Similar to higher plants, mosses also respond to environmental and nutritional factors that influence PUFA biosynthesis, including GLA, making them attractive candidates for controlled culture-based production strategies. Researchers have manipulated factors such as light intensity, temperature, CO_2_ concentration, and nutrient availability to increase GLA production in mosses. Higher light intensity and optimal temperatures can boost GLA production. Elevated CO_2_ levels can further enhance crop output, and a proper nutrient supply is essential for achieving high yields (Decker et al., 2003). Moss *Physcomitrella patens*, employing response surface methodology (RSM), in a culture medium containing sucrose (62.92 g/L), potassium nitrate (0.80 g/L), and glutamate (1.42 g/L) showed a significant increase (4.61-fold) in GLA production compared to the standard BCD medium (Chodok, 2022).

In a study, high amounts of very long-chain PUFAs, such as ARA, were reported in 7 moss species (*Physcomitrella patens, Encalypta streptocarpa, Pottia lanceolata, Plagiomnium undulatum, Atrichum undulatum, Brachythecium rutabulum, Rhynchostegium* (Mural). These species were grown under fully standardised laboratory axenic culture conditions for comparative metabolic studies. The species were modified in Knop medium under light intensity conditions of 55–70 μmol/m/s and a photoperiod of 16 h of light and 8 h of dark at 23°C. In the model organism *P. patens*, differences in fatty acid composition were observed between filamentous protonema and leaf gametophores, as determined by desaturase obtained from microarray analysis, consistent in both growth stages (Beike et al., 2014). Depending on the growth stage and species, the amount of ARA varied between 6% and 31% of total fatty acids. Spatial investigation of the corresponding *FADS* revealed the endoplasmic reticulum as the cellular compartment for ARA synthesis. The results showed that very long PUFAs are very abundant metabolites in mosses. Standard cultivation techniques using photobioreactors, combined with the availability of genome sequences and the high rate of homologous recombination in the mitotic cells of *P*. *patens,* form the basis for targeted metabolic engineering (Decker and Reski, 2012).

A research study identified and modelled the key factors that significantly affect the production of unsaturated fatty acids and biomass *P*. *patens*. The study analysed nine culture variables: temperature, agitation speed, pH, Sucrose, Diammonium Tartrate, CaCl_2_.2H_2_O, MgSO_4_.7H_2_O, KH_2_PO_4_, and KNO_3_ in a solid BCD environment using the Plackett-Burman statistical design method. The statistical analysis revealed that the moss’s pH and temperature significantly impact biomass production and the production of polyunsaturated fatty acids (PUFAs), such as linoleic acid (LA) and gamma-linolenic acid (GLA). Additionally, the study found that three nutritional variables–sucrose, CaCl_2_, and MgSO_4_ – only affected the production of specific PUFAs. Specifically, higher concentrations of sucrose were found to positively influence the production of LA, ARA, and EPA. On the other hand, higher concentrations of metal ions like CaCl_2_ and MgSO_4_ were found to hurt the production of ARA and EPA. The study concluded that pH, temperature, sucrose, CaCl_2_, and MgSO_4_ are critical parameters for the growth of *P*. *patens* and the production of PUFAs by this moss (Chodok et al., 2010). Hence, the *P*. *patens* cell suspension culture is regarded as the most suitable medium for PUFA synthesis and could be an appropriate source for producing and commercialising PUFAs. However, the results of this preliminary study on *P*. *patens* did not progress to the oil extraction stage (Chodok et al., 2010).

**FIGURE 4:**
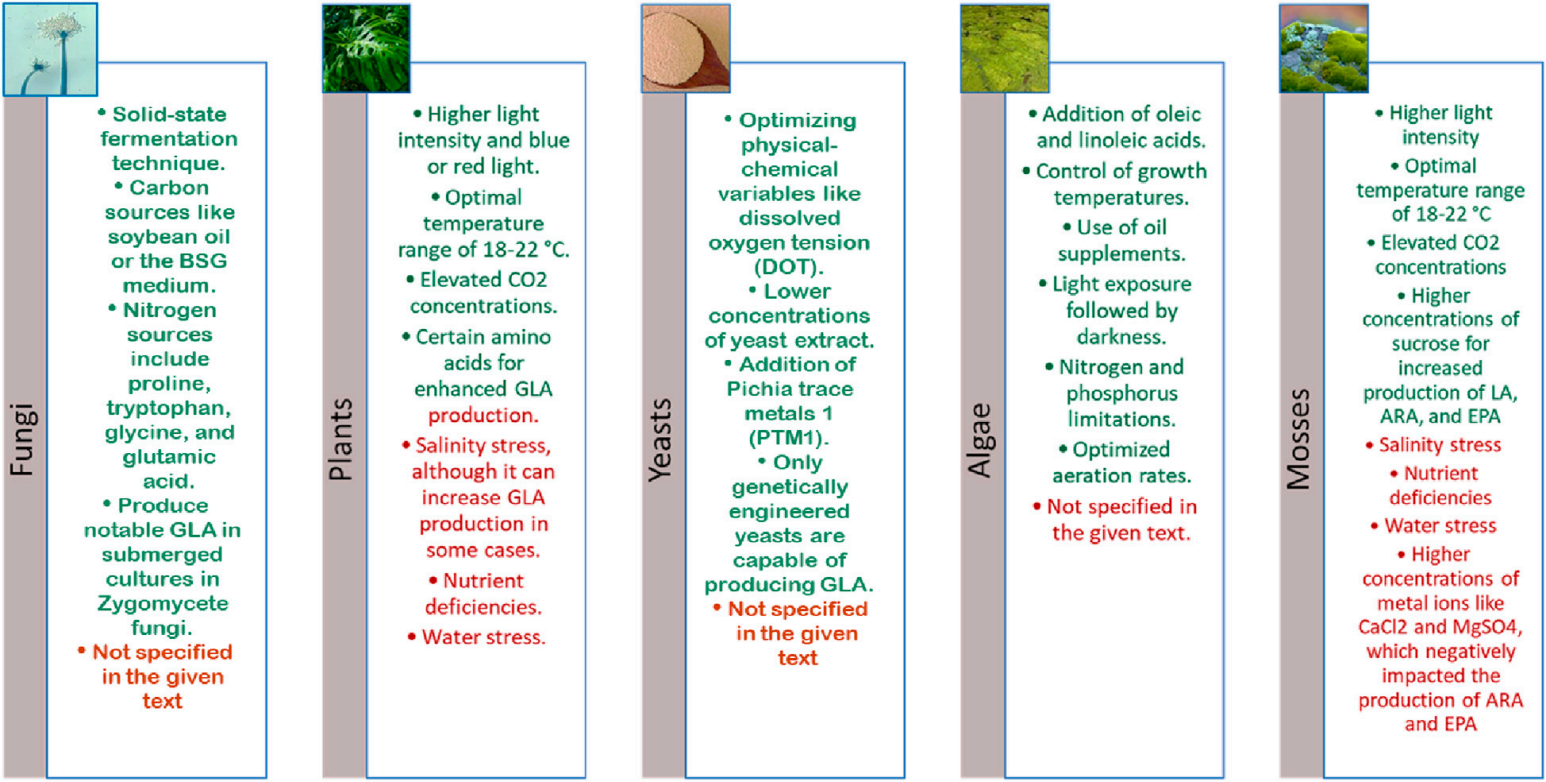
Positive (green) and Negative (red) effects of each organism. This figure summarises the key biological sources and influencing factors on γlinolenic acid (GLA) production. GLA is synthesised by certain Zygomycete fungi such as *Mucor* spp. and *Thamnidium elegans*, particularly in submerged culture systems, while edible and medicinal mushrooms do not produce GLA. In plants, environmental factors like light, temperature, CO_2_ concentration, and specific amino acids (e.g., glutamine, histidine, cysteine) enhance GLA biosynthesis, whereas salinity, water limitation, and nutrient stress may suppress it. GLA production in algae is species-dependent and influenced by factors such as light intensity, nutrient availability, and medium supplementation. Mosses show limited data on GLA biosynthesis, and exposure to stress or heavy metals tends to reduce their polyunsaturated fatty acid levels. Lastly, yeasts require genetic engineering to produce GLA, as wild-type strains lack the biosynthetic pathway. Culture conditions like pH, carbon-to-nitrogen ratio, and aeration predominantly affect lipid accumulation in yeasts.

In another study, the *D5E* gene was transferred from *Pavlova* sp. algae to *P. patens* strain Gransden to increase the production of fatty acids. For this purpose, the *D5E* gene was cloned under the control of the CaMV 35S promoter in the Gateway pMDC43 vector. Then, gene transfer was performed using polyethene glycol (PEG). The concentration of ADA and DPA increased dramatically and increased to 24.3 and 11.7 mg/L, respectively, and accounted for 1.1% and 2.3% of the total fatty acids. The obtained results prove that the pathway of fatty acid biosynthesis with genetic manipulation and food supplements is a promising method for the production of specific PUFAs in a simple seedless plant (Chodok et al., 2012). In another study, two *D12D*s associated with the early stages of long-chain PUFA biosynthesis were successfully cloned from *P. patens*, and their functions were characterised. The open reading frames (ORF) of *PpFAD2-1* and *PpFAD2-2* consist of 1,112 base pairs and code for 375 amino acids. The polypeptides obtained from them showed 62% and 64% similarity with microsomal *D12D* from higher plants, and each contained three typical histidine clusters. Examining the expression of these genes in the yeast system indicates the production of a significant amount of PUFA. In addition, decreasing the growth temperature from 30°C to 15°C increased the accumulation of unsaturated fatty acid products (Chodok et al., 2013) (Figure 4). While the genetic manipulation of mosses offers intriguing possibilities, it presents challenges in terms of cost, scalability, and efficiency compared to other host systems. Genetic modifications in mosses are typically more labour-intensive and time-consuming. However, the unique advantages of mosses, such as their high rate of homologous recombination and ability to grow in controlled environments, make them valuable for targeted metabolic engineering and PUFA production.

#### 4.3.2 Culture optimization in fungi and yeasts

More recently, research interests have been oriented toward the feasibility of some fungi species that may be able to produce a desirable PUFA named GLA. There are various fungi species, including *Umbelopsis isabellina* (Papanikolaou et al., 2004), *Cuninghamella echinulata* (Fakas et al., 2008a), *M. ramanniana* (Dyal and Narine, 2005), *M. alpina*, and *M. rouxii* (Shivaramu et al., 2008), where it is reported to produce notable levels of GLA under optimised culture conditions.

To improve GLA yield, two standard fermentation methods are utilised: SSF and submerged fermentation (SMF) (Khongto et al., 2011). SSF offers advantages such as high yields of GLA, lower production costs, reduced equipment requirements, and minimal waste generation (Maurice, 2019). It is particularly beneficial for the production of enzymes and other metabolites required for GLA synthesis. On the other hand, SMF allows for greater control over fermentation parameters, such as temperature, pH, and oxygen levels, resulting in more consistent and scalable production. Additionally, SMF facilitates better mass transfer of nutrients, contributing to more efficient fermentation processes (Belorkar and Jogaiah, 2021).

Optimisation of carbon and nitrogen sources is a significant strategy for enhancing GLA production in fungal fermentation. For example, the supplementation of soybean oil (10 mL/L) extensively promoted lipid production in *Phaffia rhodozyma*, which resulted in high levels of fatty acids like palmitic acid, stearic acid, oleic acid, linoleic acid, alpha-linolenic acid, and GLA (Li et al., 2023). Moreover, *M. alpina* and *M. circinelloides* have been reported to be the most efficient fungi for the production of GLA. *M. alpina* produced 7 g/L biomass and 900 mg/L of fatty acids, with 41% of the fatty acids being polyunsaturated, like ARA and thereby a strain of interest for the production of ARA. Conversely, *M. circinelloides* produced 6.5 g/L biomass and 600 mg/L of fatty acids, beating *Rhizopus stolonifer*, which had produced just 250 mg/L of GLA (Serra et al., 2023).

Other studies have investigated the impact of various carbon and nitrogen sources on GLA production. For instance, research on *Mucor plumbeus* with *Bacillus subtilis* bacteria found that the combination of cellobiose as the carbon source and ammonium sulfate as the nitrogen source maximised biomass, lipid, fatty acid, and GLA yield. The optimal fermentation conditions were a pH of 6.5, a temperature of 30°C, 10% inoculum, 200 rpm agitation, and a 5-day incubation period. Notably, cocultures of *M. plumbeus* and *Bacillus subtilis* produced GLA concentrations that were twice as high as those in monocultures. In addition, the role of amino acids in enhancing GLA productivity was also investigated, and results showed that strains like *Rhizomucor pusillus* AUMC i11616. A and *Mucor circinelloides* AUMC 6696 had increased GLA production when cultured in proline and tryptophan as the sources of nitrogen, with an increase of 38% and 25%, respectively, compared to an ammonium tartrate control. Glycine and glutamic acid were also shown to have significant functions as precursors of total fatty acid (TFA) synthesis (Azemi et al., 2022).

A novel technique, overexpression of mitochondrial malate transporter proteins, has also hastened lipid accumulation in *M. circinelloides*. During a 24-h fermentation period that included the addition of 0.8% malic acid (MA), recombinant strain *M. circinelloides* McMT2 yielded 16 g/L fungal dry biomass, 32% lipid content, and 5.12 g/L yield of lipids. These were 1.6 times greater in biomass and lipid content, and 2.56 times greater in lipid yield compared to the control batches not supplemented with MA.

Apart from malic acid, other carbon sources have been explored for their effectiveness in the yield of fungal lipids. For example, *M. isabellina* DSM 1414 exhibited the highest biomass and oil production of 10.80 g/L and 5.44 g/L, respectively, under lactose-based culture conditions. The major fatty acids formed under the conditions above were oleic acid (20.42%–42.94%), palmitic acid (14.96%–22.19%), stearic acid (9.00%–26.92%), linoleic acid (11.35%–18.67%), and GLA (3.56%–8.04%) (Zhang Y. et al., 2021).

Furthermore, *Rhodosporidium toruloides* AS 2.1389 was also studied in lipid production with acetic acid as a cheap carbon source. The organism demonstrated higher lipid content when grown in 4–20 g/L acetic acid than in glucose-based media. Optimal conditions with 20 g/L acetic acid and a C/N ratio = 200 resulted in 4.35 g/L biomass with 48.2% lipid content. Since there is an increased demand for natural sources of beta-carotene and C18 PUFAs, a study was conducted to assess the potential of 28 isolates of Zygomycetes, which were collected from soil. While all the fungi produced C18 PUFAs, nine were capable of producing beta-carotene. The best beta-carotene and GLA producer was *Actinomucor elegans* CCF 3218, with a yield of 251 mg/L. Conversely, *U. isabellina* CCF 2412 was the most promising strain because it had the dual potential to produce both GLA (217 mg/L) and beta-carotene (40.7 mg/L) (Huang et al., 2016; Klempova et al., 2013).

*Cunninghamella bainieri* 2A1 was also explored for its GLA and lipid production under conditions of nitrogen starvation. The highest lipid (6.2 g/L) and GLA (0.4 g/L) content were achieved using 20.2 g/L glucose and 2.12 g/L ammonium tartrate-containing media and were harvested at 48 h (Ganjali Dashti et al., 2014).

Certik et al. (2006) investigated the impact of substrate selectivity on the production of GLA in various strains of fungi that belong to the Zygomycete class. Their findings emphasised the significant role played by carbon sources in the biosynthesis of GLA, as oat flakes were found to be a superior substrate compared to peeled barley. Among the strains that were tested, *Thamnidium elegans* CCF 1465 produced the highest GLA content, at 4.82 g/kg on oat flakes but only 1.11 g/kg on peeled barley. Additionally, *Cunninghamella elegans* (CCF103 and CCF1318) and *Mucor mucedo* CCF1348 showed a tremendous growth enhancement and GLA yield when grown on oat flakes, with yields of 3.58–4.56 g/kg, while only 0.82–1.15 g/kg on peeled barley. This finding strongly suggested that oat flakes are an excellent carbon source, which favours the production of lipids in fungi (Čertik et al., 2006). In comparing the productivity of GLA in different species, *Thamnidium elegans* CCF 1,465 was the most productive strain, followed by *Mucor* and *Cunninghamella* species, which produced moderate levels. *M. isabellina* produced comparatively low GLA content, reflecting poor biosynthetic efficiency. The *Rhizopus* species (*R. oligosporus*, *R. microspores*, and *Rhizopus arrhizus*) produced consistently less than 1.0 g/kg of GLA on both substrates.

With optimised conditions, biomass yield, total lipid, and GLA yield were examined in several Zygomycetous isolates. The study highlighted the impact of varied culture conditions—pH, temperature, nitrogen, and carbon sources—on GLA productivity. *Cunninghamella blakesleeana* JSK2 isolates from Karnataka (India) soil samples were identified as the highest-yielding GLA among the ten fungi isolates evaluated. Under optimum conditions of 28°C, pH 6.0, and glucose and potassium nitrate as carbon and nitrogen sources, *C. blakesleeana* JSK2 gave 21.09% GLA of total lipids, of which 7.34% was in dry biomass. Culture condition optimisation and genetic manipulation can have beneficial roles in industrial large-scale production (Sukrutha et al., 2014).

Lipid metabolism and GLA production in *C. elegans* NRRL Y-1392 were investigated with emphasis on the role of the carbon-to-nitrogen (C/N) ratio in enhancing the storage of lipids and biomass formation. Under a high nitrogen level (C/N = 11.0 mol/mol), the fungus had excellent glycerol utilisation and produced high biomass (11.9 g/L), along with the highest GLA and PUFA production (573 mg/L and 224 mg/L, respectively). Under nitrogen starvation (C/N = 110.0 and 220.0 mol/mol), glycerol was utilised at a slower rate, but lipid biosynthesis was increased, with the highest lipid content achieved in C/N = 220.0 mol/mol (59% dry biomass). Temperature was also a determining factor in GLA production since higher temperatures (>25°C) supported growth while diminishing the GLA content. Lowering the temperature to 12°C enhanced GLA accumulation, but it also extended the culture duration and reduced the production rate. The study further identified that pH fluctuations affected fungal metabolism, with nitrogen-excess media causing acid secretion and orange colouration, indicative of an alteration in the fungal metabolic pathway. The research also evaluated the scalability of GLA production in bioreactors, attributing challenges such as biomass heterogeneity and optimisation needs of agitation and aeration parameters. Future research will focus on optimising these parameters to enhance oxygen transfer, nutrient supply, and overall efficiency, paving the way for industrial applications of GLA production (Vasilakis et al., 2024).

The ability of *C. elegans* NRRL-1393, an oleaginous fungus, to produce unsaturated fatty acids, such as gamma-linolenic acid (GLA) was investigated by Kalampounias et al. (2024a). The fungus was cultivated under nitrogen starvation in shake-flask media containing glycerol or glucose (both at approximately 60 g/L) as the sole carbon source. Both substrates supported equal amounts of biomass, about 13.5 g/L after 330–360 h of growth. However, glycerol synthesised a slightly higher amount of lipids compared to glucose. The latter produced 8.4 g/L of lipids against 7.0 g/L of glucose. The lipids contained significant amounts of unsaturated fatty acids such as oleic acid (C18:1), linoleic acid (C18:2), palmitic acid (C16:0), and GLA (C18:3) that constituted about 9.5% of the total lipids. The lipids were then saponified to yield fatty acid lithium salts (FALS), which exhibited cytotoxicity towards human cancer cell lines, indicating that they have potential as therapeutic drugs. The saponification reaction optimisation for FALS synthesis was also investigated concerning reflux time, hexane volume, and NaCl concentration. All of these parameters were found to influence the fatty acid yield significantly. The study demonstrated that FALS obtained from *C. elegans* lipids exhibited cytotoxic activity even at low doses, affirming their potential as anticancer agents. The finding suggests that FALS could enhance the efficacy of chemotherapy by inducing oxidative stress in cancer cells while minimising collateral damage to healthy cells. The combined action of oxidative stress induced by GLA and the anticancer effect of lithium holds bright prospects for therapeutic synergies.

Further research into the growth conditions revealed glycerol to be a superior carbon source for lipid storage in *C. elegans,* and nitrogen starvation to be required for maximising lipid biosynthesis. The 330–360-h growth period allowed maximum biomass and lipid yield, while extended cultivation led to a decrease in GLA productivity. Scale-up operations in bioreactors revealed that optimisation of oxygen supply, pH, and nutrient levels would increase lipid yield even more, making it industrially viable. The research also tested other substrates for fungal growth, including lipid fermentation wastewater (LFW) of *M. ramanniana*. The novel medium was utilised to carry out green SSF with fungi under nitrogen- and carbon-scarce conditions. The primary carbon source employed was glycerol, which yielded a high level of lipid accumulation coupled with the production of PUFA, particularly GLA. In glycerol-limited cultures, glycerol uptake was rapid, lipid content being at approximately 24% (w/w), and GLA accumulation at levels of up to 430 mg/L. As compared to the above, nitrogen limitation allowed greater lipid material (45% w/w), albeit GLA productivity was lower at 350 mg/L. This observation reflects a general trend in wild-type oleaginous Zygomycetes: when lipid accumulation in dry cell weight exceeds 25% (w/w), the proportion of GLA in total fatty acids tends to decrease. Conversely, under nitrogen-rich or glycerol-limited conditions, where lipid accumulation is restricted, GLA content in total lipids is relatively higher. This inverse relationship between total lipid content and GLA concentration within lipids highlights the importance of tailoring fermentation conditions to balance biomass, total lipid production, and GLA enrichment (Melanouri et al., 2024). This phenomenon may be linked to differential regulation of desaturase enzymes under nutrient-replete vs. starvation conditions.

The study also demonstrated that LFW can serve as an effective substrate for cultivating edible and medicinal mushrooms. The use of LFW promoted fungal biomass development, enhanced the activity of ligninolytic and hydrolytic enzymes, and stimulated exopolysaccharide synthesis. These findings highlight LFW’s potential for application in biotechnological processes aimed at recycling industrial waste and generating value-added byproducts.

In particular, the study explored the utilisation of LFW as maceration water in SSF systems for mushroom cultivation. Although edible and medicinal mushrooms such as *Pleurotus ostreatus* do not naturally synthesise PUFAs like GLA, ARA, or DHA, LFW proved beneficial in stimulating fungal metabolism and enzymatic activity. Adjusting the carbon-to-nitrogen (C/N) ratio significantly influenced metabolic outcomes: lower C/N ratios enhanced the activity of ligninolytic and hydrolytic enzymes, while higher ratios favoured biomass accumulation. The fruiting of *P. ostreatus* on LFW-based substrate led to the degradation of total phenolic compounds and a marked increase in laccase activity. Furthermore, high C/N conditions supported carposome formation and contributed to greater biological efficiency and yield.

While these mushrooms are not PUFA-producing organisms, the integration of LFW into their cultivation presents a sustainable and economically viable strategy for managing industrial effluents and producing bioactive fungal compounds. In contrast, microbial lipid biotechnology remains the primary pathway for PUFA production and represents a promising alternative to conventional plant-based oils.

However, the production cost of single-cell oil (SCO) remains a challenge. To mitigate this, researchers have tried to use industrial and agro-industrial waste as low-cost carbon sources. For instance, tomato waste hydrolysate (TWH) and crude glycerol have been discovered to be promising substrates for oleaginous fungi to biosynthesise lipids during nitrogen starvation conditions. Experiments with *C. echinulata* revealed the effect of limiting nitrogen on lipid content and GLA production. Cultivation of *C*. *echinulata* in TWH-supplemented medium resulted in lipid content in biomass of approximately 39.6%, and GLA content was 802 mg/L with 96–120 h of incubation. The study also demonstrated that the use of various carbon sources, such as glucose (30–90 g/L) or glycerol (30–50 g/L), influenced lipid accumulation, highlighting the strain’s versatility in cultivation on diverse substrates for microbial lipid production. Other oleaginous microorganisms, such as *Y. lipolytica* and *M. isabellina,* can convert crude glycerol into microbial lipids. *Y. lipolytica* demonstrated an extremely high lipid content of 51.7% (w/w) in biomass, further confirming its potential for large-scale production of SCO (Fakas et al., 2008b; Fakas et al., 2008a). The accumulation of lipids by the strain has also been studied using various carbon sources, including glucose and glycerol. Hence, it proved to be flexible in terms of strain regarding the application of different substrates for the production of SCO. In a different experiment, *T. elegans* CCF-1465 was used to grow in submerged shake-flask culture utilising low-cost sugar refinery wastes, *i.e*., glucose, fructose, and sucrose, as carbon sources. The fungus exhibited high lipid production, with a notable accumulation during the initial phase of sugar consumption. Sugar uptake reduced progressively while lipid accumulation continued to occur. The maximum lipid yield was more than 9 g/L with an optimum 70% (w/w) lipid content in dry biomass, suggesting the suitability of the strain for large-scale lipid production. The lipids produced by the microbes were largely oleic and palmitic acids and seemed suitable as feedstock for biodiesel fuel. GLA was also produced at a level of approximately 510 mg/L, once more highlighting the potential for the utilisation of *T. elegans* in GLA production (Papanikolaou et al., 2010). Tables 6 and 7 contain additional information regarding the various conditions used in fungi in GLA production.

**TABLE 6:** Media engineering and physical conditions in different organisms.

Organism	Media engineering	Physical condition	References
*Mucoraceous* fungi	Carbon sources, such as glucose, sucrose, starch, and lactose, influenced lipid and GLA production in these fungal cultures Grown for 4 days at 28°C, shaking at 150 rpm, the maximum fungal biomass for AUMC 6696. A was 14.6 ± 0.2 g/L with arginine and 13.68 ± 0.1 g/L with asparagine (as single nitrogen sources), while AUMC 11616. The maximum biomass was 10.73 ± 0.8 g/L with glycine and 9.44 ± 0.6 g/L with valine	Low temperatures (25°C), vigorous light intensity (6 Klux), and primrose oil supplementation (0.8% w/v) induced high GLA accumulation in these strains	Somashekar et al. (2003), Ronda and Lele (2008), Sukrutha et al. (2014)
*Cunninghamella* sp	The culture was maintained on potato dextrose agar (PDA) plates at 4 °C and transferred to PDA plates every 3 weeks	It was grown at 28°C for 2 days and then stored at 4 °C until fermentation	Song et al. (2022)
*Mucor plumbeus*	Efficiency is achieved at 6.5 pH, 30°C, 10% (v/v) inoculum composition, 200 rpm agitation speed, and a 5-day incubation period		Mohamed et al. (2022b)
*Umbelopsis isabella*	Cultivation was performed at 28°C for 5 or 7 days, and the obtained bioproducts were collected and dried at 65°C until a constant weight was achieved		Slaný et al. (2020)

**TABLE 7:** GLA production in wild-type and genetically modified fungal systmes

Species	Cultivation method	Carbon source	Nitrogen source	Temp (°C)	pH	Duration	SCO composition	References
*Cunninghamella echinulata* ATHUM 4411	Rotary shaker, Erlenmeyer flasks, Agitation at 180 rpm	Xylose, Raw glycerol, Glucose	(NH_4_)_2_SO_4_, Yeast extract	28	5.3–6	360h	16.7% GLA 1,119 mg/L	Fakas et al. (2009)
*Mortierella isabellina* ATHUM 2935	Rotary shaker, Erlenmeyer flasks, Agitation at 180 rpm	Xylose, Raw glycerol, Glucose	(NH_4_)_2_SO_4_, Yeast extract	28	5.3–6	360h	4.1% GLA 250 mg/L	Fakas et al. (2009)
*Cuninghamella echinulata* ATHLM4411	Batch. flask	Glucose	Orange peel	28	4.1–6.4	480 h	16.4% GLA 720 mg/L	Gema et al. (2002)
*Aspergillus* sp*.* ATHUM 3482	Submerged fermentation (Batch mode, orbital shaker at 200 ± 5 rpm)	Waste cooking olive oil (15 g/L)	(NH_4_)_2_SO_4_ (5 g/L), Yeast extract (0.5 g/L)	28	4.5–6	Variable	<0.6% GLA	Papanikolaou et al. (2011)
*Aspergillus niger (A. niger* NRRL 363*, A. niger* NRRL 364*, A. niger* LFMB 1*, A. niger* LFMB 2*)*	Submerged fermentation (Batch mode, orbital shaker at 200 ± 5 rpm)	Waste cooking olive oil (15 g/L)	(NH_4_)_2_SO_4,_ (5 g/L), Yeast extract (0.5 g/L)	28	4.5–6	Variable	<0.6% GLA	Papanikolaou et al. (2011)
*Thamnidium elegans*	SSF	Cereal materials (wheat bran, spelt wheat flakes, oat flakes, spent malt grains)	Cereal materials (wheat bran, spelt wheat flakes, oat flakes, spent malt grains)	24	6	96 h	13%–14% ≈1,422 mg/L Wheat bran/SMG(3:1)	Certik et al. (2012)
*Mortierella ramanniana* MUCL 9235	Orbital shaker, 185 rpm	Glycerol (30 g/L) or Glucose (30 g/L)	(NH_4_)_2_SO_4_, Yeast Extract (0.5 g/L)	28	5–6	142 h	200 mg/L (biodiesel-derived glycerol)	Papanikolaou et al. (2017)
*Cunninghamella echinulata* LFMB 5	Orbital shaker, 185 rpm	Glycerol (30 g/L) or Glucose (30 g/L)	(NH_4_)_2_SO_4_, Yeast Extract (0.5 g/L)	28	5–6	336 h	405 mg/L (glucose)	Papanikolaou et al. (2017)
*Mortierella isabellina* ATHUM 2935	Orbital shaker, 185 rpm	Glycerol (30 g/L) or Glucose (30 g/L)	(NH_4_)_2_SO_4_, Yeast Extract (0.5 g/L)	28	5–6	155–222 h	216 mg/L (biodiesel-derived glycerol), 50 mg/L (glucose)	Papanikolaou et al. (2017)
*Mortierella ramanniana*	Flask culture	Glycerol (35 g/L)	Glycerol (≈35 g/L)	26	6		GLAmax ≈430 mg/L (9%–13%) under carbon limitation, GLAmax ≈350 mg/L (6%–9% g/L) under nitrogen limitation	(Melanouri et al., 2024)
*Cunninghamella echinulata* CCF-103	Shake flask culture, 180 rpm	Glucose (70 g/L)	Corn gluten, corn steep, whey concentrate yeast extract, tomato waste hydrolysate	28	6	96–120 h	11.5% (802 mg/L) Tomato waste hydrolysate	Fakas et al. (2008a)
*Cunninghamella echinulata* ATHUM 4411	Shake flask culture, 180 rpm	Glucose (30–90 g/L) or Glycerol (30–50 g/L)	Organic nitrogen from tomato waste hydrolysate (TWH)	28	5.5–6	168 h	1,018 mg/L	Fakas et al. (2008b)
*Thamnidium elegans* CCF-1465	Submerged shake-flask culture, orbital shaker at 200 rpm	Glucose, Sucrose, Fructose (30, 60, 80 g/L)	(NH_4_)_2_SO_4_ (0.5 g/L), Yeast extract (0.5 g/L)	29	5.3–5.8	96 h	510 mg/L	Papanikolaou et al. (2010)
*Cunninghamella blakesleeana* JSK2	Shake flask, 120 rpm	Glucose, Sucrose, starch (30 g/L)	Potassium nitrate (1 g/L), yeast extract (5 g/L)	24–32	4–8	Different duration (2,4,6,8 days)	21% ≈ 220.2 mg/L 120 rpm for 6 days with optimum pH 6°C and 28°C	Sukrutha et al. (2014)
*Mucor rouxii* CFR*-*G15	Shake flask, rotary shaker (200 rpm)	Glucose (65 g/L)	Yeast extract (3.5 g/L), Ammonium nitrate (0.5 g/L)	28	6	144 h (6 days)	18.55% ≈692 mg/L	Mamatha et al. (2008)
*Thamnidium elegans* TE7*-*15	Fermentation	raw glycerol	(NH_4_)_2_SO_4_, Yeast extract				1.08 g/L (1,079.95 mg/L)	Ling et al. (2000)
*Thamnidium elegans* CCF-1465	Shake flask (250 mL, 50 mL working volume) and bioreactor (3 L, 1.5 L working volume)	Glucose (100 g/L), Xylose (100 g/L), Glucose–Xylose mixtures (50:50, 75:25, 25:75 g/L)	Yeast extract (4 g/L), (NH_4_)_2_SO_4_ (2 g/L)	28	>5.2–6.2	309 h	1,014 mg/L GLA	Zikou et al. (2013)
*Cunninghamella elegans* NRRL Y*-*1392	Batch flask and Bioreactor (3 L and 20 L)	Glycerol (30–50 g/L)	Yeast Extract (Y.E.) + Ammonium Sulfate (A.S.)	12, 20, 28	6	108–480 h	12.8% (243 mg/L) in 108 h. 28°C 15.5% (310 mg/L) in 480 h. 12°C	Vasilakis et al. (2024)
*Umbelopsis isabellina* CCF2412	SSF	Wheat bran, cornmeal, animal fat (animal fat as a carbon source)	(NH_4_)_2_SO_4_, Yeast extract	28	4–5.4	168 h (7 days)	6.4 mg GLA/g of FBM ≈640 mg/L	Slaný et al. (2021)
*Actinomucor elegans*	SSF	Apple pomace (waste biomass)		25–30		288 h (12 days)	3.85 g GLA/kg DW of pomace ≈385 mg/L	Dulf et al. (2023)
*Umbelopsis isabellina*	SSF	Apple pomace (waste biomass)		25–30		288 h (12 days)	4.34 g GLA/kg DW of pomace ≈434 mg/L	Dulf et al. (2023)
*Rhizomucor pusillus* AUMC 11616.A	Shake flask, 150 rpm, K&R medium with amino acid nitrogen source	Glucose (80 g/L)	proline	28	4–8	96 h	13.4% ≈ 376 mg/L	Mohamed et al. (2022a)
*Mucor circinelloides* AUMC 6696.A	Shake flask, 150 rpm, K&R medium with amino acid nitrogen source	Glucose (80 g/L)	Tryptophan	28	4–8	96 h	12.8% ≈ 431 mg/L	Mohamed et al. (2022a)
*Thamnidium elegans*	Submerged fermentation	Glucose (90 g/L)	(NH4)_2_SO_4_	28	5–6	550 h	371 mg/L	Chatzifragkou et al. (2011)
*Mortierella isabellina*	Shake flask, nitrogen-limited conditions	Glucose (45–100 g/L)	, Yeast extract (0.5 g/L each)	28	6	250 h	4.4% 801 mg/L	Papanikolaou et al. (2004)
*Umbelopsis isabellina*	SSF	Grape pomace	NaNO_3_ (4 g/L), Yeast extract (1 g/L)	28	6	288 h	7.35% 3.79 mg/100 g pomace	Dulf et al. (2020)
*Actinomucor elegans*	SSF	Grape pomace	NaNO_3_ (4 g/L), Yeast extract (1 g/L)	28	6	288 h	4.16% 1.93 mg/100 g pomace	Dulf et al. (2020)
*Mortierella (Umbelopsis) isabellina* ATHUM 2935	Batch flask, nitrogen-limited	Glucose and xylose (80 g/L) or glucose/xylose mixtures (60:20, 40:40, 20:60)	Yeast extract (0.5 g/L) + (NH_4_)_2_SO_4_ (0.5 g/L)	28	5.2–6	524 h	5.41% ≈600 mg/L	Gardeli et al. (2017)
*Mortierella ramanniana* MM15-1	Agitation-aeration in jar-fermentor	Glucose (300 g/L)	Urea (7.8 g/L)	26	5	216 h (9 days)	≈17% ≈5,500 mg/L	Hiruta et al. (1997)
*Cunninghamella echinulata* CCRC 31840	Shake flask, 200 rpm, 250-mL flask with 25-mL medium	Soluble starch (100 g/L)	NH_4_NO_3_ (1.1 g/L), Yeast extract (5 g/L)	25		120 (5 days)	11.7% 1,349 mg/L	Chen and Liu (1997)

The research on yeast culture engineering for GLA production involves genetic manipulations and optimisation of fermentation processes to enhance GLA yields (Gu et al., 2023). In the study by Khongto et al. (2011), a fed-batch fermentation technique was used to increase the production of GLA in *Hansenula polymorpha*. After optimising physical-chemical variables during fermentation, the researchers found that the most critical factor influencing GLA output was Dissolved Oxygen Tension (DOT). The ideal parameters for generating GLA were DOT saturation at 28%, yeast extract at 1 g/L, and *Pichia* trace metals 1 (PTM1) at 3.6 mL/L. GLA content was higher in low quantities of yeast extract and lower in higher concentrations. These optimised conditions improve scalability for industrial production by enhancing yeast growth and lipid accumulation, making large-scale GLA production more feasible (Khongto et al., 2011).

#### 4.3.3 Culture optimisation in algae

The research investigated how *Spirulina platensis* produces GLA in different stressful conditions. The experiments demonstrated that adding oleic acid and LA at various growth temperatures, along with oil supplements, enhanced the production of fatty acids. Also, when the cultures were exposed to varying light levels, the ratio of GLA to total fatty acids remained constant. The best conditions for producing lipids and GLA in algae depend on various factors such as temperature, light intensity, salinity, and specific oil supplements. Studies suggest controlling these factors is crucial for enhancing GLA production in algae cultures (Wang et al., 2022; Jin et al., 2019). Specific parameters for GLA production in algae, including temperature, light intensity, salinity levels, and oil supplements, have also been identified. Optimising these parameters can improve GLA production in algae, making them suitable for industrial applications (Table 8).

**TABLE 8:** Effective parameters in algae culture optimisation.

Parameter	Effect	References
Temperature	Low temperatures around 25°C have been found to induce high GLA production in *S. platensis*, with a yield of 13.2 mg/g of dry cell weight	Ronda and Lele (2008)
Light Intensity	The light solid intensity of 6 Klux has also been shown to increase GLA production, yielding 14.6 mg/g in *S. platensis* cultures	Ronda and Lele (2008)
Salinity	Optimum salinity levels, such as 5 g/L, have been identified to enhance GLA productivity in marine green algae, resulting in a 1.6-fold increase in GLA productivity	Miura et al. (1993)
Oil Supplements	Specific oil supplements, like primrose oil at 0.8% (w/v), have been shown to stimulate high GLA accumulation in cyanobacteria, with a maximum GLA yield of 13.5 mg/g of dry cell weight	Ronda and Lele (2008)

Spirulina, a commercial cyanobacterium, has been suggested as a more affordable source of GLA, a dietary supplement that is currently expensive. Studies have shown that Spirulina contains PA, saturated fatty acids, LA, and GLA. Spirulina also has the highest percentage of omega-6 fatty acids at 18.8 g. The production of highly pure GLA from microalgal sources could be a promising approach due to their high oil production, fast reproduction, and non-food nature (Choopani et al., 2016).

Higher GLA content can be achieved by manipulating light conditions, such as growing algae under light and then leaving them in the dark. Nitrogen and phosphorus limitation are influential factors for increasing lipid content. Nitrogen increases total fat, while phosphorus limitation significantly affects fat profile, and this type of commercial production of GLA from spirulina can be achieved by shortening the harvest time (6–8 days) and optimising aeration rates. The most straightforward and most efficient method to obtain GLA concentrate is urea accumulation, which does not require any organic solvent except ethanol. The recommended method for lipid extraction from Spirulina on an industrial scale is a three-step process: supercritical extraction, ethanol extraction, and aqueous extraction (Choopani et al., 2016). GLA production is regulated by phosphate and nitrate levels during growth and temperature control. Antarctic cyanobacteria can act as a source of GLA (Verma et al., 2021). Optimising culture conditions (light, salinity and temperature) in algae impacts the cost-effectiveness and sustainability of industrial GLA production.

Algae-based GLA production offers specific advantages over other organisms, like yeast or fungi. Algae are more environmentally sustainable because they can quickly absorb CO_2_ and produce high amounts of biomass. Additionally, algae do not compete with food crops for arable land, making them an economically viable option for large-scale production. Algae can be cultivated in various environments, including wastewater, reducing production costs and environmental impact.

## 5 Therapeutic and clinical applications of GLA

The pharmaceutical industry has shown considerable interest in GLA’s therapeutic potential. It has been studied for managing conditions such as diabetic neuropathy, rheumatoid arthritis, and atopic dermatitis (Chang et al., 2017). GLA’s anti-inflammatory and neuroprotective properties have been the basis for developing GLA-based drugs and supplements to treat various health issues (Pagels et al., 2022). GLA is an omega-6 fatty acid with various potential health benefits. GLA has been shown to reverse diabetic neuropathy, a severe complication of diabetes caused by high blood sugar levels that damage the nerves (Pop-Busui et al., 2022). Diabetic animals and humans cannot convert dietary LA to GLA, which is necessary for standard neuronal structure and function (Ramli et al., 2017). Supplementing helps correct biochemical defects and restores GLA metabolite levels, significantly improving clinical symptoms and neurophysiological function in diabetic neuropathy (Shi and Zhao, 2017). In a controlled clinical trial involving 111 patients with mild diabetic neuropathy, supplementation with 480 mg of GLA per day for 1 year resulted in notable improvements in 13 out of 16 evaluated neuropathy parameters. The beneficial effects were observed regardless of factors such as gender, age, type, or duration of diabetes. However, the treatment had a more significant impact on patients whose diabetes was relatively well-controlled (Horrobin, 1992). Another study involving 22 patients with diabetic distal polyneuropathy found that daily intake of 360 mg of GLA for 6 months led to significant improvements in the signs of neuropathy compared to the placebo group (Peng et al., 2022). These findings suggest that supplementation can effectively improve symptoms, including nerve conduction velocity, sensory nerve function, motor activities, tendon reflexes, and sensory functions in the hands and feet of patients with diabetic neuropathy (Hernandez, 2016). At the same time, GLA may control blood lipids by reducing triglyceride levels, total cholesterol, and LDL cholesterol while increasing HDL cholesterol. In a clinical trial involving 12 patients with hyperlipidemia, the daily intake of 3 gr of GLA for 4 months resulted in a 48% reduction in plasma triglyceride levels, a 22% increase in HDL cholesterol levels, and a significant decrease in total cholesterol and LDL cholesterol (Sun et al., 2017). These findings suggest that GLA supplementation has a positive impact on lipid profiles and improves cardiovascular health. However, further research is necessary to fully understand the effects on blood lipids and their clinical significance (Won et al., 2020). Additionally, GLA supplementation has been found to help improve symptoms of irritable bowel syndrome (IBS), such as abdominal pain, bloating, diarrhoea, constipation, and cramping (Keen et al., 1993). GLA achieves its cardiovascular effects predominantly through its anti-inflammatory action, which is mediated by its beta-oxidation to DGLA and subsequently to bioactive eicosanoids such as PGE_1_ and 15-HETrE (as outlined in Section 4.1). These cause vasodilatation, inhibit platelet aggregation, stabilise blood pressure, and improve lipid profiles, thereby reducing the risk of arteriosclerosis and vascular dysfunction (Nilsen et al., 2021; Bertoni et al., 2023). Due to its immunomodulatory effects, GLA is also being proposed as an adjunctive treatment for inflammatory disorder-related conditions, such as arthritis and atopic dermatitis (Ruiz-López et al., 2012). By enhancing PGE_1_ production, it may reduce NSAID reliance by reducing joint stiffness and inflammatory signs (Chen et al., 2010; Tański et al., 2022). The processes mentioned above, already explained in the biosynthesis of GLA section, point towards the systemic benefits of GLA-derived metabolites for cardiovascular and inflammatory regulation.

When used in conjunction with NSAIDs, GLA acts as a synergistic agent and prevents common gastrointestinal side effects associated with NSAIDs (Sohail et al., 2023). Additionally, GLA inhibits leukotriene synthesis and cytokine production, demonstrating benefits in diseases such as asthma and acute respiratory distress syndrome (ARDS) (Su et al., 2023). Rheumatoid arthritis (RA) is an autoimmune disease characterised by immune system attacks on the body’s tissues, resulting in joint inflammation and cartilage destruction. Common symptoms include joint pain, swelling, and stiffness, which can lead to long-term damage, chronic pain, and disability (Leventhal et al., 1993). Clinical trials confirm GLA’s immunoregulatory and anti-inflammatory properties, highlighting its synergistic potential when used alongside standard therapies in inflammatory and autoimmune diseases (Zhang C. et al., 2021; Zurier et al., 1996). Another double-blind, placebo-controlled clinical trial involved 56 patients with active rheumatoid arthritis. They received either 8.2 gr of GLA or a Placebo (sunflower oil) daily for 6 months. Following this phase, all patients received treatment for an additional 6 months in a single-masked manner. The therapy led to a substantial decrease in disease activity (Furse et al., 2001). Atopic dermatitis, a chronic inflammatory skin disorder commonly referred to as eczema, is characterised by symptoms such as dry skin, itching, and redness. It is associated with a D6D deficiency, which converts LA to GLA. Recent studies have shown that supplementation with GLA, found in evening primrose oil, can help alleviate skin inflammation and reduce associated symptoms (Horrobin, 2000). In clinical trials, patients who consumed evening primrose oil experienced a decrease in eczema severity scores. Increasing plasma GLA levels can also be a predictive marker for the response to evening primrose oil treatment. Therefore, evening primrose oil is an effective and safe option for managing atopic dermatitis (Williams, 2003). Multiple Sclerosis (MS) is a neurological condition characterised by inflammation and damage to the central nervous system. Some theoretical evidence suggests that GLA may be beneficial in MS. Animal studies have shown that GLA suppresses the activity of the immune system, particularly by inhibiting the proliferation of T lymphocytes, and reduces the severity of MS-like disease (Kousparou et al., 2023). Researchers from New Zealand have observed that MS patients often experience cold hands and feet, which is typically a sign of impaired peripheral blood flow. An uncontrolled study conducted on 16 patients demonstrated that GLA from evening primrose oil improves peripheral circulation and consequently enhances the strength and function of the hands and feet in these patients. The high content of essential fatty acids also reduces inflammation associated with MS-related neurological damage.

Recent studies have significantly enhanced knowledge about GLA and its anticancer effect, especially when synthesised in microorganisms. Conventionally, research has been focused on “pure” GLA of plant or chemical origin. More and more, however, the focus has shifted to microbial GLA, which is considered “impure” because it is part of the overall lipid in microorganisms. Various studies have linked microbial GLA with diverse cancers, viz., leukaemia, glioblastoma, pancreatic cancer, breast cancer, prostate carcinoma, and papillary thyroid carcinoma. Various journal studies have established that microbial GLA induces oxidative stress, triggers apoptosis and ferroptosis, inhibits cell proliferation, and reduces cell migration and viability, thus indicating its therapeutic value against cancer (Kalampounias et al., 2024a; Kalampounias et al., 2024b; Kairemo et al., 1998).

In addition, recent research has also highlighted that GLA can be obtained from fungi, specifically those of the Zygomycota phylum, which can produce high concentrations of GLA when cultivated on low-cost feedstocks such as glycerol. This production is an economic and environmentally friendly alternative to genetically engineered strains for the mass production of GLA. A specific fungus, *Cunninghamella elegans* NRRL1393, has been demonstrated to produce high concentrations of lipids when cultured on glycerol, about 8.4 g/L. The GLA-rich lipids from this fungus were then transformed into fatty acid lithium salts (FALSs). The FALSs were found to be cytotoxic with an IC_50_ value of approximately 60 μg/mL, potently inhibiting cell migration, inducing oxidative stress, and leading to cell death in normal human and cancer cell lines. These findings suggest a general, non-specific mechanism of action, and the high level of oxidative stress may enhance the effectiveness of traditional anticancer therapies (Kalampounias et al., 2024b).

Besides fungi, it was also found to be able to produce PUFAs, including GLA, which are linked with anticancer properties. Such fungi, when cultivated on glucose or glycerol as a carbon source, produce lipids that can be converted into FALS. The produced FALSs have been found to exhibit good potential to inhibit the growth and movement of prostate cancer cell lines, DU145 and PC3, making them more susceptible to lipid derivatives. In contrast to the olive oil-derived FALSs when employed as a control, the microbial FALSs drastically inhibited the proliferation and migration capacity of the cancer cells. These results suggest that microbial lipids, particularly those of fungi such as *T. elegans* and *M. alpina*, possess potential anticancer properties in suppressing the growth and motility of cancer cells (Kalampounias et al., 2024a).

Additional therapeutic potential was also demonstrated by an investigation of the polyunsaturated lipids produced by *Thamnidium elegans* grown on raw glycerol and the microalga *Nannochloropsis salina*. The fungal lipids, which were GLA-rich, consisted mainly of neutral lipids (82%). These lipids were converted into fatty acid potassium salts (FAPS), which potently inhibited the growth of several bacteria, including Gram-negative bacteria such as *Serratia* sp. and *Neisseria gonorrhoeae*, as well as Gram-positive bacteria like *Staphylococcus aureus* and *Enterococcus faecalis*. Furthermore, the FAPS were highly toxic against the MCF7 cancer cell line at very low concentrations. These findings suggest that FAPS of microbial polyunsaturated lipids could be developed as novel therapeutic medications (Sayegh et al., 2016).

This building evidence attests to the therapeutic potential of microbial GLA, showing not only that it performs well in isolated forms but also in its derivatives from microbial lipids. Fungal lipids as a source of GLA provide a cost-effective, scalable, and viable approach to cancer drug production.

Evening primrose oil, rich in GLA, is a beneficial natural supplement for women’s health. It relieves premenstrual syndrome (PMS) symptoms by increasing the production of anti-inflammatory prostaglandins, thereby easing muscle cramps and bloating. During menopause, it provides a natural alternative to hormone replacement therapy (HRT), effectively reducing hot flashes, mastalgia, inflammation, and other discomforts by balancing eicosanoids. It also helps manage cyclical breast pain with fewer side effects than medications and significantly reduces hot flash frequency and intensity in postmenopausal women, enhancing their quality of life (Hu et al., 2021). Table 9 illustrates the therapeutic use of GLA in humans.

**TABLE 9:** Examples of GLA’s therapeutic use in humans.

Illness	Sex	Dose	Efficiency	Mechanism	References
Rheumatoid arthritis	Male/Female	540 mg GLA/day for 48 weeks	Reduced or stopped using NSAIDs to control their symptoms after 12 months	Inactivation of STAT3 pathway Inhibition of the production of inflammatory cytokines in RAFLS and CIAN rat models	Hajiaghayi et al. (2024), Zhang et al. (2021a)
Diabetic neuropathy	Male/Female	320 mg GLA/600 mg ALA daily for 12 weeks	Improving neurological symptoms by increasing the speed of transmission of motor nerve messages, the function of sensory nerves, combined muscle activities, tendon reflexes, and sensory functions in the hands and feet	Antioxidative and vasodilatory effects	Ziegler, 2023; Won et al. (2020)
Hypercholesterolemia	Male/Female	5 mg/kg for 28 days	Improved TC, TG, HDL, and LDL levels in a high-fat-diet-induced hypercholesterolemic rat model		Azemi et al. (2022)
Cyclic mastalgia	Female	evening primrose oil 500 mg capsules twice daily	Reduce breast pain		Seifalyazal et al. (2023)
Pregnancy discomfort	Pregnant women	1,000 mg (twice daily) of the oral evening after the 35th week	Preventing poisoning in the final stages of pregnancy and delaying childbirth, facilitating childbirth	Evening primrose oil, with prostaglandins E_1_ and E_2_, has a calming effect on smooth muscle Prostaglandins affect cervical dilation and contraction by changing the tone and consistency of cervical blood vessels	Poor et al. (2023), Mahboubi (2019)
PMS	Female	180 mg/day	Stimulation of prostaglandin synthesis relieves symptoms of PMS		Gandhi and Desai (2022)
Eczema	Female/Male	loading the membranes with 10 µL blackcurrant seed oil rich in 11%–19% of GLA	Effective treatment of skin symptoms	Improve skin condition and skin texture Elevation of blood concentrations of catecholamine	Sroczyk et al. (2022)
Cancer	Female/Male	1–5 mg/day for 7–10 days (direct intratumoral injection)	Regression of human gliomas without any significant side effects, Antiangiogenic action	Modulation of steroid hormone receptors A valuable adjunct to primary tamoxifen in endocrine-sensitive breast cancer	Das and Das (2020), Kenny et al. (2000), Lupu et al. (2012)

Current studies suggest that a deficiency in certain fatty acids may make individuals more susceptible to COVID-19. Specifically, low levels of GLA, DGLA, ARA, eicosa pentaenoic acid (EPA), and increased lipid peroxidases and free radicals are associated with severe COVID-19 cases. Higher rates of mortality from COVID-19 are observed in patients with diabetes, hypertension, and coronary artery disease, which may be attributed to their deficiency in GLA, DGLA, ARA, and alpha-linolenic acid (ALA) (Das, 2021). Conversely, the lower prevalence of GLA and milder cases of COVID-19 in children and women may be associated with higher levels of specific fatty acids such as BioActive Lipids (BALs). Children are believed to have a higher capacity for producing fatty acids such as GLA and DGLA, which can be converted into beneficial compounds that combat SARS-CoV-2. Premenopausal women produce significant amounts of LXA4 (lipoxin A4), which confers resistance and reduces the likelihood of severe COVID-19 (Tong et al., 2019). Research indicates that PUFAs, such as GLA and DGLA, act as antibacterial, antifungal, antiviral, and immunomodulatory agents. They can inhibit the entry of coronaviruses and other enveloped viruses into target cells by modulating the fluidity of cell membranes and regulating the ACE-2 receptors responsible for viral entry. PUFAs, including GLA, also modulate the production of M1 and M2 macrophages, reducing the production of pro-inflammatory cytokines like IL-6 and TNF-α. Therefore, they can treat severe cytokine storms in COVID-19 (Matschinske et al., 2020; Baral et al., 2022).

## 6 Industrial applications

**FIGURE 5:**
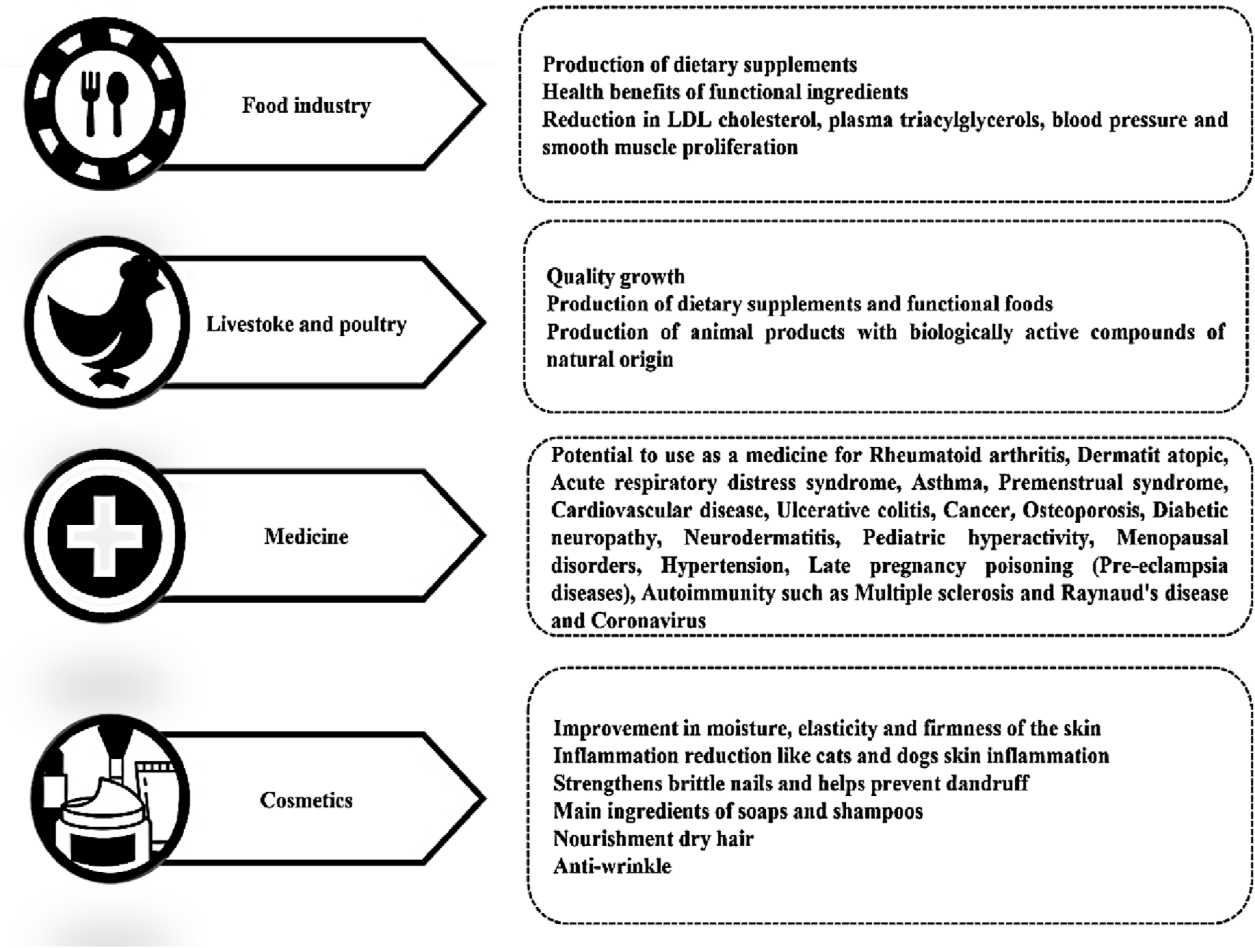
GLA Applications in various industries.

GLA has a range of applications (Figure 5) in the food, cosmetics, livestock, poultry, and pharmaceutical industries (Su et al., 2023). Within the food sector, GLA is valued for its nutritional benefits, particularly its ability to promote heart health and reduce inflammation (Khan et al., 2019). Functional foods enriched with GLA are being developed to enable consumers to incorporate this beneficial ingredient into their everyday diet (Kaur et al., 2014). Methods to enrich food products with GLA and other polyunsaturated fatty acids (PUFAs) include direct addition, microbial supplementation, microencapsulation, or feeding animals PUFA-rich diets (Fan YangYi and Chapkin, 1998). In Japan, *Mortierella isabellina* is used to produce GLA-rich lipids, which are then utilised as food additives and functional dietary supplements (Demir and Gündes, 2020).

The demand to increase the fatty acid content of animal products, such as meat and eggs, for their improvement in therapeutic and nutritional value for human consumption has accelerated in recent years. Supplementation of animal diets with GLA is one possible method of improving quality of animal products, along with animal health and resistance (Sergeant et al., 2016; Navarette et al., 1992). Due to the limited enzymatic conversion of LA to GLA in animals and humans, dietary supplementation with GLA-enriched feed ingredients has grown as an intervention (Lin et al., 2013).

Various feed supplements containing PUFA, including fish oil, flaxseed, rapeseed, and borage oil, have been tested to determine whether they can raise the levels of GLA in animal tissues. Of these, borage oil has been of therapeutic importance in the management of inflammatory disorders such as ovine chronic interstitial pneumonia and serves as a functional nutrient in equine and ruminant nutrition (Casas-Cardoso et al., 2021). Borage plant supplementation has also been proposed as a stress-mitigating feeding regimen for herbivores during periods of physiological transition (Montaner et al., 2021). Apart from vegetative feedstocks, fungal fermentation through oleaginous Zygomycota fungi is an emerging alternative for the production of GLA-enriched feedstocks, particularly by fermenting crop residues such as grains and glycerol (Chang et al., 2010).

Aside from its applications in animal medicine, GLA has also gained significant interest in the pharmaceutical, cosmetic, and nutraceutical industries. GLA is a highly sought-after ingredient in the cosmetics and personal care industry due to its beneficial effects on skin health. It is known for its moisturising properties, anti-inflammatory effects, and suitability for treating a variety of skin conditions (Gu et al., 2023). GLA-rich oils are commonly incorporated into products such as creams, serums, lotions, soaps, and shampoos to hydrate the skin, delay ageing, and address issues like dry skin, eczema, acne, and skin sensitivity (Kapoor and Nair, 2005). It enhances skin hydration, elasticity, and firmness while combating wrinkles and rough skin texture. Furthermore, its anti-inflammatory properties and ability to regulate sebum production make it effective in treating acne (Ruiz-López et al., 2012). GLA supplements and oils also help alleviate skin problems, allergies, redness, and itching. It plays a crucial role in skin care and promoting healthy skin and hair (Sergeant et al., 2016).

Due to the various applications of GLA, the demand for pure and cost-effective production methods has increased. As a result, various extraction and purification methods have been developed to purify GLA from plant and microbial sources. Traditional solvent extraction methods using hexane or ethanol remain common in the case of seed oils of plant species (e.g., evening primrose, borage), but are normally plagued by poor yield, purity, and environmental issues. To make up for the inadequacies, newer methods such as supercritical CO_2_ extraction, enzymatic hydrolysis, and urea complexation have been utilized to selectively extract and purify GLA fractions. These technologies are able to increase purity of GLA greater than 80%, and are especially beneficial for downstream applications that require high-grade raw material (Sayegh et al., 2016). Moreover, enzymic interesterification and structured lipid production have been utilized in the incorporation of GLA into omega-3-enriched triglycerides to enhance end product nutritional value and bioavailability. These developments are essential in the design of formulated lipids for clinical nutrition, dermatology, and cardiometabolic therapy. Commerically, the worldwide market of GLA has experienced consistent growth. This increasing trend is the result of increasing consumer awareness of functional fatty acids, along with general industrial requirements for natural, plant, and microbial lipid products. Major players such as Sonova, Efamol, and Nature’s Way have been leaders in the commercialization of GLA-enriched products. Sonova concentrates on high-purity, bioavailable oils for the control of inflammation and immune support. Efamol incorporates GLA with antioxidants and vitamins in its formulations for skin wellness and hormonal stability. Nature’s Way, on the other hand, offers multi-ingredient formulas that are suitable for various nutritional and therapeutic needs, particularly in the women’s health and cardiovascular segments.

Overall, the industrialisation of GLA, including biotechnological production and purification, through to market inclusion, asserts its growing significance as a multifunctional lipid. Continued work to streamline extraction protocols, ensure regulatory compatibility, and integrate GLA into functional ingredients will enhance its position in the global market, opening up opportunities for sustainable and health-enhancing bioactives.

## 7 Challenges and future directions

Several key challenges persist in hindering the large-scale microbial production of GLA, despite recent advances in metabolic engineering and culture optimisation. These challenges cross biotechnological, genetic, and regulatory fronts. These must be addressed to fully exploit the industrial and therapeutic potential of GLA-producing microbial platforms. A comparative overview of GLA yields in wild-type and genetically modified organisms is presented in Table 10. This table illustrates how significant improvements in GLA yields are achieved through metabolic engineering strategies, including gene overexpression, pathway rerouting, and strain optimisation. These comparisons highlight the inherent significance of genetic modification in overcoming the native constraints of wild-type strains and achieving scalable, commercially viable production outcomes.

**TABLE 10:** Comparison of GLA production in wild-type and genetically engineered organisms.

Organism	Examples	GLA yield	Cost-effectiveness	Environmental sustainability	Industrial scalability	Advantages	Disadvantages
Plants	*Oenothera biennis*, *Ribes nigrum*, *Borago officinalis*, *Cannabis sativa*	8%–25% of total fat (species-dependent)	Low to moderate (seasonal, variable quality)	Moderate (requires land, water, and specific climate)	Moderate (limited by agricultural constraints)	Agricultural potential, rich natural GLA sources, well-characterised genetics	Seasonal growth, low productivity, time-consuming extraction, low acceptance of GM plants
Oleaginous fungi	*Mortierella* spp., *Cunninghamella* spp., *Mucor* spp.	Up to 30% of total fat (species-dependent)	High (can use cheap substrates like glycerol)	High (no land needed, can use waste substrates)	High (scalable via fermentation)	Rapid growth, modifiable fatty acid profiles via culture conditions, high PUFA content	High bioreactor cost, complex and labor-intensive purification
Microalgae	*Spirulina platensis*, *Neochloris oleoabundans*	18%–21% of total fat	Low to moderate (slower growth, complex harvesting)	High (photosynthetic, CO_2_-utilizing, no land needed)	Low to moderate (harvesting and processing challenges)	Short harvest cycles, high photosynthetic efficiency, disease resistance	Unappealing for food, genetic engineering limitations
Engineered yeasts	*Yarrowia lipolytica*, *Pichia pastoris*, *Saccharomyces cerevisiae*	4.6%–22.5% of total fat	Moderate (requires bioreactors, but fast growth)	High (can use various substrates, eco-friendly)	High (well-suited for industrial-scale fermentation)	Easy genetic manipulation, GRAS organisms (e.g., *Y. lipolytica*), use of cheap carbon sources, no endotoxins	High cost of fermentation infrastructure Requires precise culture control
Mosses	*Physcomitrella patens*	Low (less than 5% of total fat)	Moderate (cheap media and simple growth conditions)	High (small footprint, eco-compatible)	Moderate (high genetic engineering potential, limited yield)	High efficiency of homologous recombination, Advanced genetic tools, rapid growth in simple systems	Low GLA content, requires genetic improvement for industrial use

### 7.1 Technical and bioprocess bottlenecks

Microorganisms, particularly oleaginous fungi and yeasts, offer a promising and scalable system for GLA production. However, large-scale production of the fatty acid faces several bioprocess, genetic, and engineering challenges that limit the full exploitation of its therapeutic and commercial potential.

The first significant challenge is the limitations of scalability in fermentation processes. For instance, in the 1980s, large-scale cultivation of *Mucor circinelloides* for GLA-rich single-cell oil (SCO) production was successfully demonstrated in large stirred-tank bioreactors at an industrial scale. This resulted in biomass with 25% total lipids, of which up to 19% was GLA. Yet, difficulties related to bioreactor design, oxygenation, and even nutrient supply in dense cultures led to inefficiencies on an industrial scale (Ratledge, 2013). The other significant challenge in microbial GLA production is the inefficiency of genetic tools. Unlike *Y. lipolytica*, which facilitates genetic modification with ease, the majority of filamentous fungi lack strong promoters, selectable markers, or stable gene delivery systems. This significantly limits the metabolic engineering of these organisms, resulting in a reliance on model strains (Sun et al., 2017).

From a bioprocess perspective, process maintenance of fermentation and real-time process monitoring optimisation at the industrial level represents another challenge. More specifically, monitoring of parameters such as lipid accumulation, pH, and dissolved oxygen remains technically challenging and typically requires expensive equipment (Belorkar and Jogaiah, 2021). In addition, substrate inconsistency represents another challenge; although the use of inexpensive substrates, such as glycerol or agricultural waste, can yield economic benefits, their unstable composition can undermine reproducibility.

Another key limitation is the formation of unwanted by-products such as ethanol, organic acids, or surplus biomass, which lowers overall GLA production efficiency. Additionally, genetic stability of the engineered strains is a concern because some strains lose their desirable phenotype after several generations, necessitating continuous monitoring and re-engineering (Gu et al., 2023).

Briefly, to surmount these issues, interdisciplinary solutions incorporating genetic engineering, next-generation bioreactor design, the development of real-time monitoring systems, and the use of optimised and homogeneous feedstocks are required. By using technologies such as synthetic biology, intelligent control systems, and GRAS-status microbial platforms, GLA biosynthesis productivity can be transformed.

### 7.2 Regulatory and social constraints of GMOs

Genetically modified organisms (GMOs) are increasingly employed for GLA production due to their capacity to express desaturase genes, optimise metabolic fluxes, and improve lipid yields. For instance, transgenic *Yarrowia lipolytica* strains have reached a maximum of 22.5% GLA content in total fatty acids (Sun et al., 2017), while transgenic safflower seeds, which were transformed with delta-6 desaturase, have attained over 40% GLA content (Nykiforuk et al., 2012). However, despite such advancements, the commercial application of GMOs is controlled rigorously.

Other nations, such as the European Union, Japan, and those in South America, have strict regulatory frameworks that include severe biosafety, allergenicity, and gene flow tests. These long approval processes result in marketplace delays and increased production costs. Furthermore, the public’s lack of confidence in products produced from GMOs—particularly in the nutraceutical and food sectors—continues to be a deterrent, with labelling regulations and consumer sentiment often discouraging market adoption (Huesing et al., 2016).

### 7.3 Agronomic challenges in plant cultivation

Borage, one of the most traditional sources of GLA, is also affected by several agronomic challenges that keep it small-scale as well as economically unviable (Montaner et al., 2021). Although over 95% of global borage production is concentrated in five countries—the United Kingdom, Canada, the Netherlands, New Zealand, and Poland—the crop is extremely labour-intensive to cultivate. Additionally, seed yields are variable, primarily due to premature seed fall and non-synchronous flowering, ranging from 100 to 500 kg/ha, depending on the location (El Hafid et al., 2002). Another major challenge is the poor competitiveness of borage with weeds, especially dicotyledonous weeds. This is exacerbated by a lack of herbicide-resistant crop varieties and the phytotoxicity of current herbicides, *such as* yellowing and stunting under changing environmental conditions. These constraints pose a major impediment to achieving uniform yields. Despite all this, recent progress in plant biotechnology has come as some relief. Tissue culture, somatic embryogenesis, and liquid-based systems of *in vitro* propagation have been explored to produce GLA from borage somatic embryos. These present novel avenues for biotechnological production of GLA, particularly by alleviating field-level variability and genetic control over oil traits.

### 7.4 Future directions and opportunities

To address these limitations and improve GLA productivity, the following strategies should be undertaken: integrating synthetic biology, integrating synbiotics such as CRISPR/Cas9, utilising modular cloning vectors, and employing biosensors to control pathways, which can significantly enhance production (Kan et al., 2022). Host Optimisation: the construction of GRAS-status hosts, such as *Y. lipolytica*, *P. patens,* or algae with improved stress tolerance and enhanced precursor flux, is a viable option (Gu et al., 2023). Fermentation process development, which involves improving scalability and consistency, can be achieved through the development of advanced fed-batch or continuous fermentation systems with real-time control and sensor-based monitoring (Belorkar and Jogaiah, 2021). Regulatory harmonisation and effective communication of GMOs at the global level, along with improved public communication, are necessary to ensure consumer confidence and streamline approval processes. Overcoming these technical and regulatory challenges is key to unlocking the full biotechnological and commercial value of microbial GLA production. Multidisciplinary solutions that bring together strain engineering, process optimisation, and regulatory change must be mobilised to bring sustainable, high-productive, and socially acceptable GLA biofactories to market.

## 8 Conclusion and discussion

Gamma-linolenic acid (GLA) is a valuable dietary polyunsaturated fatty acid with demonstrated health virtues in humans, particularly for the management of inflammation, cardiovascular disease, and metabolic disorders. Due to the continuously increasing demand worldwide for GLA, scientists have been attempting to discover novel methods for enhancing production, maximising extraction methods, and determining alternative sources apart from traditional plant oils such as evening primrose and borage. One of the most promising areas for the production of GLA is microbial fermentation using oleaginous fungi such as *Mortierella ramanniana*, *Cunninghamella elegans*, *Cunninghamella echinulata*, and *T. elegans*. These accumulate lipids in high amounts, and some strains can produce over 1,000 mg/L of GLA when subjected to optimised fermentation conditions. Among the fermentation processes, submerged fermentation (SmF) is the most effective microbial system for GLA production, as it allows controlled conditions of aeration, pH, and nutrient feeding. The use of glycerol as a carbon source in the glycolytic pathway under SmF has resulted in maximised lipid production. Additionally, agricultural and food processing industry wastes are being studied as low-cost and environmentally friendly substrates for fermentation. Next to oleaginous fungi, microalgae like *Spirulina platensis* have been recognised as sustainable natural sources of GLA, although less productive than fungi and consequently less efficient for industrial cultivation. Oleaginous yeasts like *Y. lipolytica* have also been recognised as novel platforms for GLA production. The organism enjoys faster growth rates compared to filamentous fungi and can be easily genetically transformed for enhanced lipid accumulation. As a result of metabolic engineering, *Y. lipolytica* can produce more GLA today and is a potential candidate for industrial fermentation. In parallel, genetic engineering plays a crucial role in enhancing the yield of GLA. Researchers have managed to increase yield by overexpressing key enzymes such as delta-6 desaturase, which is responsible for catalysing the transformation of LA into GLA. For example, GLA content has been attained at 40% of total lipids in genetically engineered *M. alpina*. Similarly, *C. echinulata* strains have been optimised to produce more lipid yield and enzymatic activity. Besides microorganisms, some plants have also been genetically transformed to synthesise GLA. The *D6D* gene has been expressed in plants such as tomato, tobacco, and canola. High GLA-content seed oil transgenic lines, such as safflower (*Carthamus tinctorius*, up to 40% GLA) were developed.

A second significant field in GLA manufacturing involves the maximisation of extraction and purification processes. Reactions such as hydrolysis and urea complexation can increase the concentration of GLA in oils by up to 40%–80%. Enzymatic and chemical modifications, such as interesterification, are also used to alter the fatty acid composition of GLA-laden oils according to their intended end-use functions. Another method is the manufacturing of structured lipids by adding GLA to omega-3-rich triacylglycerols, thereby upgrading the nutritional and functional qualities of these oils. As the commercialisation of GLA continues to rise, rigorous quality control and safety regulations have become mandatory. Sophisticated spectroscopic, chromatographic, and molecular techniques such as PCR are employed to analyse the safety and purity of GLA-rich oils. New high-throughput screening technologies, automation of bioprocessing, and lipidomics tools will significantly enhance both the efficiency of production and the quality of the product.

In the years to come, the future of GLA production will depend on the convergence of new biotechnological tools, optimisation of industrial processes, and adoption of economically beneficial and environmentally friendly production systems. The principal research fields include optimising fermentation conditions, breakthroughs in genetic engineering, and improving microbial strains through synthetic biology. Emerging technologies such as CRISPR gene editing, novel bioreactor design, and high-accuracy lipidomics profiling will transform the efficacy of GLA production and recovery. Automation systems for high-throughput screening are important in identifying top-performing strains for industrial applications, *esp*. in engineered fungi and yeast. Furthermore, the combination of microbial fermentation, plant biotechnology, and advanced extraction and purification technology will reduce production costs and increase product purity. This interdisciplinarity option not only ensures sustainable and scalable GLA availability but also opens new frontiers for its clinical, nutritional, and industrial applications. Pursuing these directions, GLA can become a key compound in future worldwide efforts at health, prevention, and nutrition.

## Abbreviations

ALA, Alpha-linolenic acid; EPA, Eicosapentaenoic acid; MS, Multiple Sclerosis; NSAIDs, Non-steroidal anti-inflammatory drugs; FAPS, Fatty acid potassium salts; FALS, Fatty acid lithium salts; D12D, Delta-12 desaturase; D5D, Delta-5 desaturase; D6D, Delta-6 desaturase; PUFA, Polyunsaturated fatty acid; DCW, Dry cell weight; OA, Oleic acid; ARA, Arachidonic acid; DGLA, Dihomo-gamma-linolenic acid; LA, Linoleic acid; GLA, Gamma-linolenic acid; SSF, Solid-state fermentation; SMF, Submerged fermentation.
